# Prognostic analysis of tumor mutation burden and immune infiltration in hepatocellular carcinoma based on TCGA data

**DOI:** 10.18632/aging.202811

**Published:** 2021-04-04

**Authors:** Shaoqing Liu, Qianjie Tang, Jianwen Huang, Meixiao Zhan, Wei Zhao, Xiangyu Yang, Yong Li, Lige Qiu, Fujun Zhang, Ligong Lu, Xu He

**Affiliations:** 1 Department of Minimally Invasive and Interventional Radiology, Sun Yat-Sen University Cancer Center, State Key Laboratory of Oncology in South China, Collaborative Innovation Center for Cancer Medicine, Guangzhou 510060, China; 2 Zhuhai Interventional Medical Center, Zhuhai Precision Medical Center, Zhuhai People's Hospital Affiliated with Jinan University), Jinan University, Zhuhai 519000, China; 3 Department of Pharmacy, Guangdong Women and Children Hospital, Guangzhou 511400, China

**Keywords:** hepatocellular carcinoma, tumor mutation burden, The Cancer Genome Atlas, immune infiltration

## Abstract

In order to explore the prognosis of tumor mutation burden (TMB) and the relationship with tumor infiltrating immune cells in hepatocellular carcinoma (HCC), we downloaded somatic mutation data and transcriptome profiles of 376 HCC patients from The Cancer Genome Atlas (TCGA) cohort. We divided the samples into high-TMB and low-TMB groups. A higher TMB level indicated improved overall survival (OS) and was associated with early pathological stages. One hundred and nine differentially expressed genes (DEGs) were identified in HCC. Moreover, based on four hub TMB-related signatures, we constructed a TMB Prognostic model (TMBPM) that possessed good predictive value with area under curve (AUC) of 0.701. HCC patients with higher TMBPM scores showed worse OS outcomes (p < 0.0001). Moreover, DCs subsets not only revealed higher infiltrating abundance in the high-TMB group, but also correlated with worse OS and hazard risk for high-TMB patients in HCC. Meanwhile, CD8+ T cells and B cells were associated with improved survival outcomes. In sum, high TMB indicates good prognosis for HCC and promotes HCC immune infiltration. Hence, DCs and the four hub TMB-related signatures can be used for predicting the prognosis in HCC as supplements to TMB.

## INTRODUCTION

Hepatocellular carcinoma (HCC) is the most common pathological subtype of liver cancer; it is the sixth most common type of cancer and the fourth leading cause of cancer-related death in the world [1–3]. Although the main treatment of early HCC is surgery, 50% of the patients are at an advanced stage at the time of diagnosis [4]. The 5-year overall survival (OS) rate for HCC is about 12.5% [3, 5]. Targeted therapy has improved the OS in HCC; however, the overall efficacy is unsatisfactory. The emergence of immunotherapy has identified new therapeutic prospects for HCC [6], especially immune checkpoint inhibitors (ICIs), such as programmed death 1 (PD-1)/programmed death ligand-1 (PD-L1) and cytotoxic T-lymphocyte associated antigen-4 (CTLA-4) [7, 8]. In recent clinical studies, both nivolumab and pembrolizumab have exhibited better prognosis as second-line therapy in advanced HCC after sorafenib treatment [9, 10].

Although ICIs therapy has been shown to be effective in several types of malignancies [11–15], it has shown varying effects in various types of cancer, even in the same cancer patients [15]. Hence, the identification of accurate biomarkers is an urgent need for screening patients who can benefit from immunotherapy and to monitor the prognosis of immunotherapy. Recently, an increasing number of biomarkers have been identified for predicting the efficacy of immunotherapy, including DNA damage repair (DDR) [16], microsatellite instability (MSI) [17, 18], neoantigens [19], and HLA presentation of neoantigens against tumor [20–22]. TMB is a novel biomarker that is calculated as genetic variations per million bases of the encoded genome [23, 24]. Patients with a high TMB have a superior objective response rate (ORR) and prolonged OS [19, 25, 26] than those with a low TMB. ORR differences can be explained by TMB in about 55% types of tumor [20]; however, TMB is not a single biomarker for predicting the efficacy of immunotherapy that may be inconsistent with specific genetic mutations in high-TMB patients. For example, patients with EGFR mutations and ALK rearrangements (EGFR+/LK+), JAK1 mutations, or JAK2 mutations are associated with low response to immunotherapy [27–30]. Meanwhile patients with KRAS mutations (KRAS+) had a better ICIs response rate [27], and STK11 mutation was found to be the main factor for PD-1 inhibitor resistance in lung adenocarcinoma with KRAS+ [31]. Moreover, tumor microenvironment (TME) has been well identified as a molecular determinant in many cancers. TUMEH [32] found that T cell infiltration in TME is closely related to the efficacy of immunotherapy. Moreover, higher infiltrating abundance of CD8+ T cell and memory activated CD4+ T cell subsets revealed prolonged OS in the high-TMB group [33]. Therefore, combined information from TMB levels, gene mutations, and immune infiltration density can be used as a novel biomarker for predicting the efficacy of immunotherapy in HCC.

The prognostic role of TMB and the relationship between TMB and immune infiltration varied for different types of cancers [33, 34], and limited studies have focused on TMB with immune infiltration in HCC. Thus, we investigated the prognostic role of TMB and the potential association between immune infiltration and hub TMB-related signature in HCC, using TCGA HCC cohort and Gene Expression Omnibus (GEO) datasets. We found that high TMB was a good prognostic predictor for HCC, and that DCs and the four hub TMB-related signatures could also be used for predicting the prognosis in HCC.

## RESULTS

### Genome-wide mutation profiling in HCC

**Figure 1:**
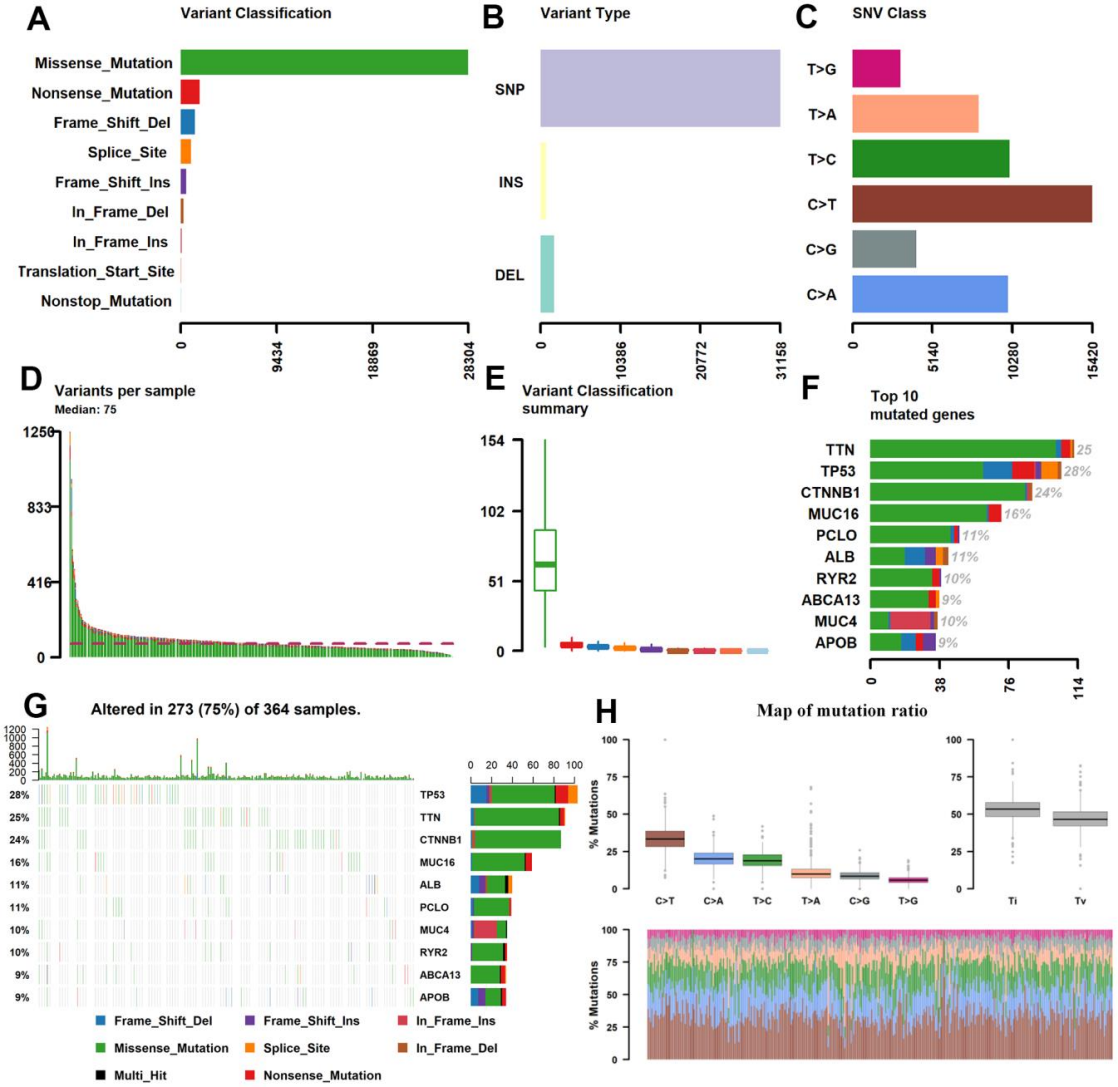
**Statistics of mutation information in the HCC samples.** (**A**–**C**, **H**) Statistical results of the different mutations, in which missense mutation occupied the most mutation classifications, SNP accounted for the main mutation type, and C>T was the main SNV Class. (**D**, **E**) Statistics of tumor mutations in each sample and different colors represent the different mutation types as shown in Figure 1A. (**F**) Statistics of different mutations in the top 10 hyper abrupt genes and different colors represent different mutation types. (**G**) The mutation status of the top 10 hyper abrupt genes: the X-axis is the sample, the Y-axis is the hyper abrupt gene, and different colors represent different mutation types. HCC, hepatocellular carcinoma; SNP, single nucleotide polymorphism; SNV, single nucleotide variants.

The somatic mutation data of 376 HCC patients were processed from the TCGA (https://tcga-data.nci.nih.gov/tcga/) database and their clinical information has been presented in Table 1. The mean age was 61 years; 122 (32.4%) women and 254 (59.5%) men were included. Utilizing maftools software, we classified these mutations into various groups and exhibited mutation groups in box plots using various colors in box plots (Figure 1). The most common type was missense mutation (Figure 1A); single nucleotide polymorphism occurred more frequently than deletion (DEL) or insertion (INS) (Figure 1B), and C>T transition was the most common form of single nucleotide variants (SNV) in HCC (Figure 1C). The mutation categories are shown in box plots (Figure 1E). The top 10 mutated genes were TP53 (28%), TTN (25%), CTNNB1 (24%), MUC16 (16%), ABL (11%), PCLO (11%), MUC4 (10%), RYR2 (10%), ABCA13 (9%), and APOB (9%) (Figure 1F, 1G).

**Table 1:** Clinical data of 376 patients with hepatocellular carcinoma (HCC) from the cancer genome atlas (TCGA) cohort in this research.

**Level**	**Overall**
N	376
Age (median [IQR])	61.00 [51.00, 69.00]
Gender (%)	
female	122 (32.4)
male	254 (67.6)
Status (%)	
Alive	243 (64.6)
Dead	132 (35.1)
Not Reported	1 (0.3)
pathologic_T (%)	
T1	185 (49.5)
T2	94 (25.1)
T3	81 (21.7)
T4	13 (3.5)
TX	1 (0.3)
pathologic_N (%)	
N0	257 (68.5)
N1	4 (1.1)
NX	114 (30.4)
pathologic_M (%)	
M0	272 (72.3)
M1	4 (1.1)
MX	100 (26.6)
pathologic_stage (%)	
Stage I	175 (49.7)
Stage II	86 (24.4)
Stage III	86 (24.4)
Stage IV	5 (1.4)
tumor_stage (%)	
stage i	175 (46.5)
stage ii	86 (22.9)
stage iii	86 (22.9)
stage iv	5 (1.3)
Unknown	24 (6.4)

### TMB was related to overall survival and clinical stage

**Figure 2:**
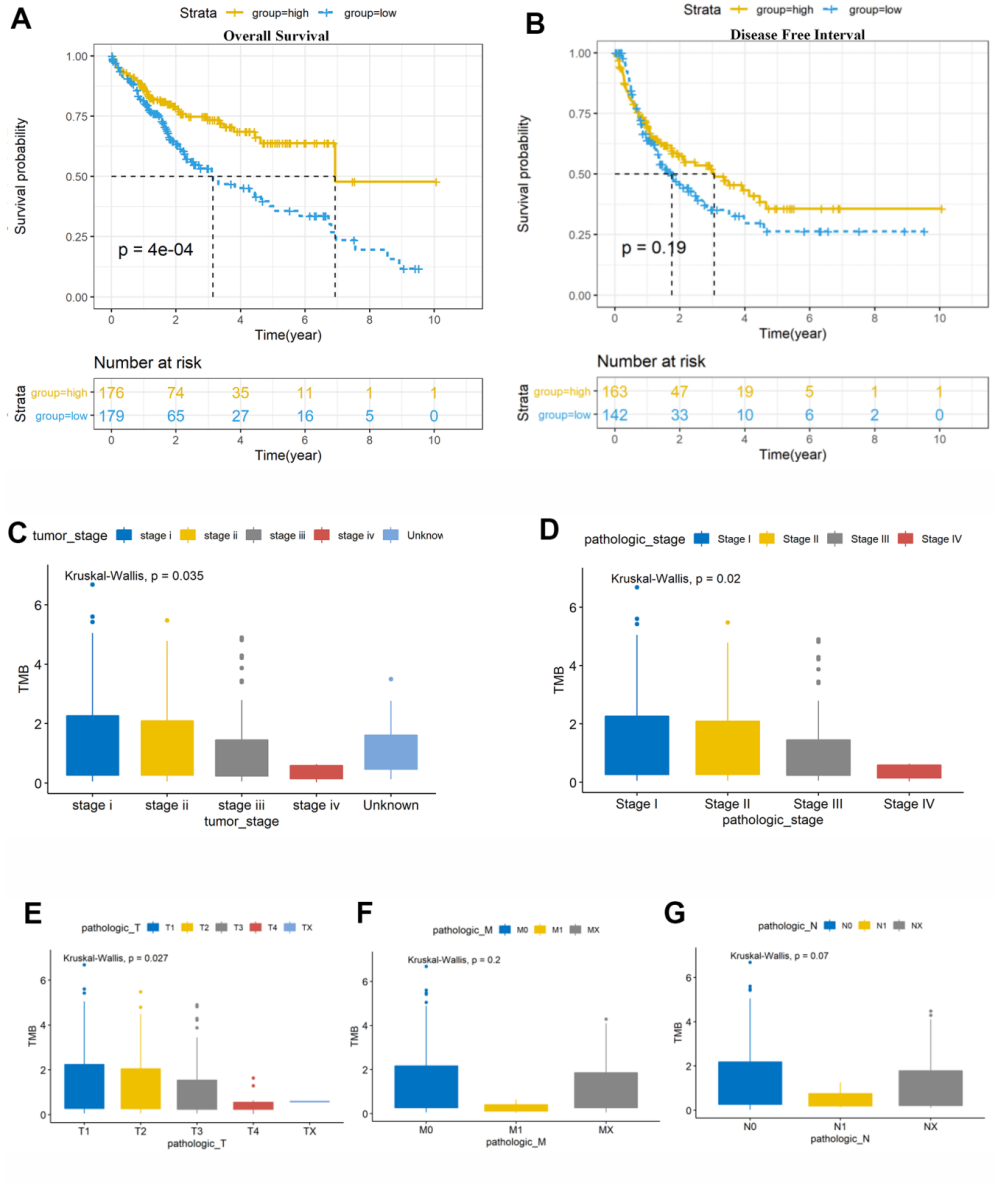
**6Prognosis analysis of tumor mutation burden (TMB) and correlation analysis with clinical risk features.** (**A**) Patients with higher TMB had better overall survival (OS, P = 0.0014). (**B**) There was no association of TMB with disease-free survival (DFS, P = 0.51). (**C**, **D**) High TMB level was negatively correlated with tumor stage and pathological stage, with P = 0.035 and 0.02, respectively. Vertical and horizontal axes represent TMB value and different stages, respectively. (**E**–**G**) Significant difference was observed in the AJCC-T stages (P = 0.027), while no significant differences were observed in the AJCC-N and AJCC-M stages (P > 0.05). TMB, tumor mutation burden. Vertical and horizontal axes represent TMB value and different stages, respectively. TMB, tumor mutation burden; OS, overall survival; DFI, disease-free interval.

We calculated the number of TMB per million bases for 363 samples and classified them into high-TMB and low-TMB groups using the median value as the threshold (Supplementary Table 1). Kaplan–Meier survival indicated that higher TMB was associated with better OS (p = 0.0004) (Figure 2A). However, high TMB was not in accordance with better disease-free interval (DFI) in this research (Figure 2B). Moreover, we found that higher TMB was also associated with tumor stage (p = 0.035; Figure 2C), pathologic stage (p = 0.020; Figure 2D), and T stage (p = 0.027; Figure 2E). The TMB levels tended to decrease with tumor progression (Figure 2C–2E), and clinical research with a larger sample size is required to verify this result.

### Analysis of differentially expressed genes between the 2 TMB groups

**Figure 3:**
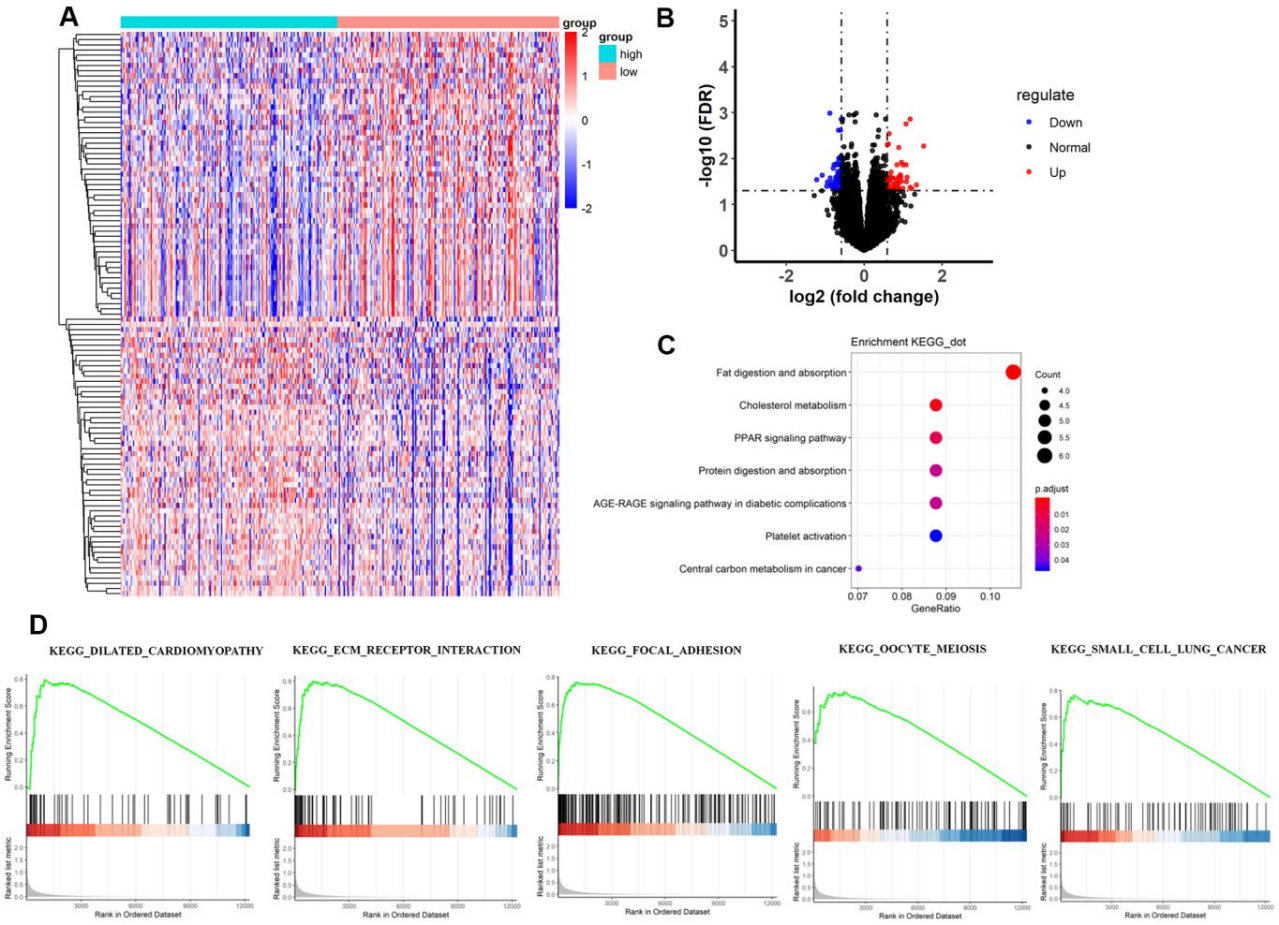
**Comparisons of gene expression profiles in low-TMB and high-TMB groups and enrichment pathway analysis.** (**A**) Top 109 DEGs are shown in the heatmap plot. Vertical and horizontal axes represent genes and HCC samples respectively, as ranked by TMB value. Genes with higher and lower levels are shown in red and blue, respectively. Color bars at the top of the heat map represent sample types, with pink and blue indicating low- and high-TMB samples, respectively. (**B**) Volcano plot of all DEGs were drawn with |log(FC) > 1| and FDR < 0.05. Each symbol represents a gene, and red, blue and black colors indicate upregulated, downregulated and normal genes, respectively. (**C**) KEGG pathway analysis revealed that these genes were involved in immune-related pathways, such as cholesterol metabolism; (**D**) Moreover, GSEA analysis shown that the top TMB-related crosstalk, including dilated cardiomyopathy, ECM-receptor interaction, focal adhesion, oocyte meiosis, and small cell lung cancer with FDR < 0.3. The vertical axis represents enrichment score. The enrichment score increased with the number of enriched genes and vice versa. DEGs, differentially expressed genes; TMB, tumor mutation burden; KEGG, Kyoto Encyclopedia of Genes and Genomes; ECM, extracellular matrix; HCC, hepatocellular carcinoma; GSEA, gene set enrichment analysis.

**Figure 4:**
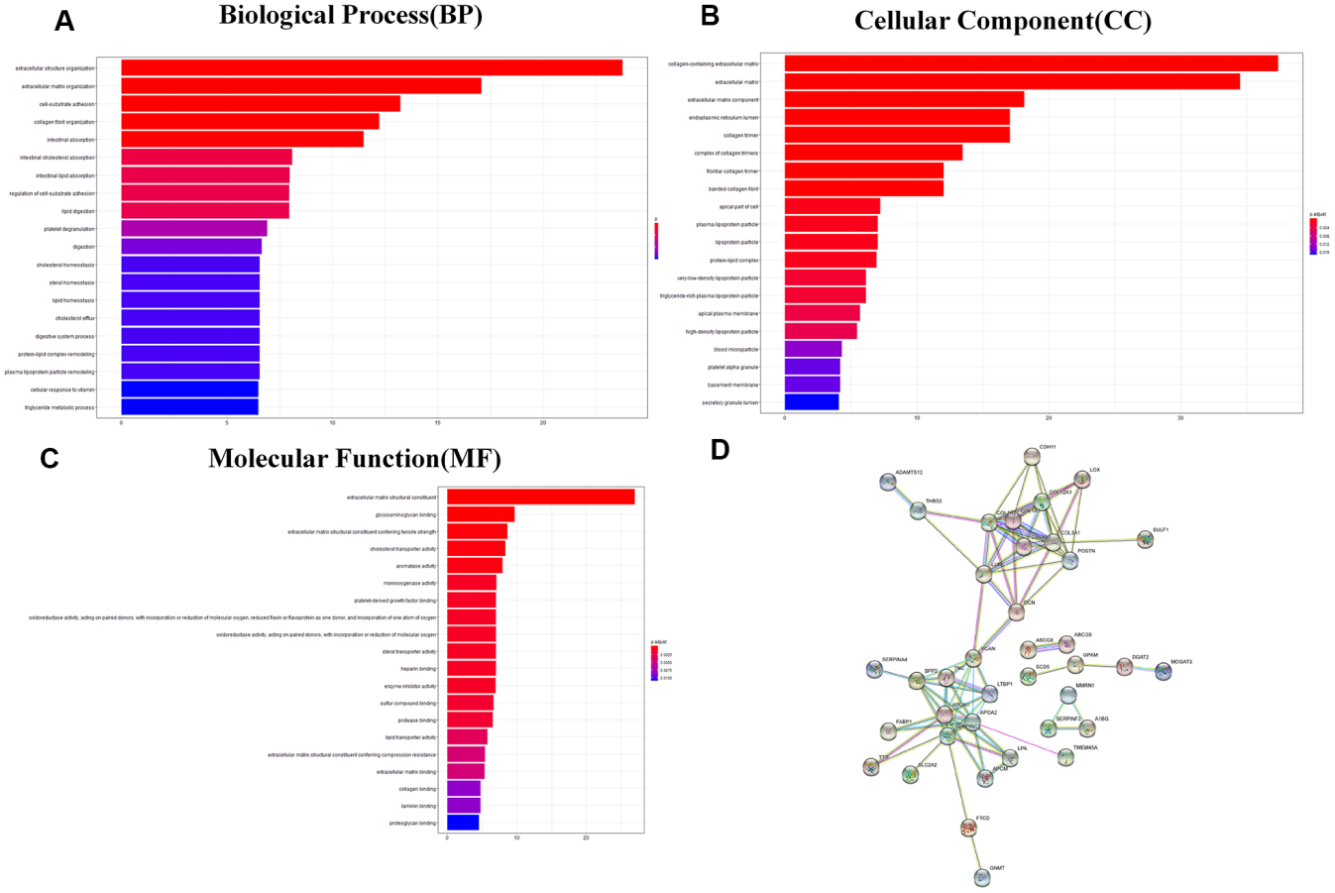
**GO analysis and protein–protein interaction (PPI) analysis.** (**A**–**C**) GO enriched results revealed that these DEGs were involved in the biological process, molecular function, and cellular component and other functional pathways. Metascape bar graph to view the top twenty non-redundant enrichment clusters. The enriched biological processes were ranked by p value. A deeper color indicates a smaller p-value. Vertical axes represent different pathway. (**D**) Thirty-six proteins were related in the protein–protein interaction. GO, Gene Ontology; PPI, protein–protein interaction; DEGs, differentially expressed genes.

The heatmap and volcano plot visualized that 109 differentially expressed genes (DEGs) were identified with limma software with |logFC|> 1.5 and false discovery (FDR) < 0.05 (Figure 3A, 3B and Supplementary Table 2). The Gene Ontology (GO) enrichment analysis demonstrated that in molecular function group, these DEGs were mainly involved in extracellular matrix structural constituent, glycosaminoglycan binding, and extracellular matrix structural constituent conferring tensile strength. In the biological process group, extracellular structure organization, extracellular matrix organization, and cell-substrate adhesion were enriched. In addition, in the cellular component, TMB-related DEGs were mainly involved in collagen-containing extracellular matrix, extracellular matrix, and extracellular matrix component (Figure 4A–4C and Supplementary Table 3). Thereafter, we conducted Kyoto Encyclopedia of Genes and Genomes (KEGG) pathway analysis and found that TMB-related signatures were involved in several pathways, including fat digestion and absorption, cholesterol metabolism, and peroxisome proliferators-activated receptor (PPAR) signaling pathway (Figure 3C and Table 2). We then selected the top gene set enrichment analysis (GSEA) results of TMB-related items, wherein dilated cardiomyopathy, ECM-receptor interaction, focal adhesion, oocyte meiosis and small cell lung cancer were associated with TMB levels (Figure 3D). Moreover, we obtained protein–protein interaction (PPI) networks with the STRING tool, and 36 proteins were related (Figure 4D).

**Table 2:** Kyoto encyclopedia of genes and genomes (KEGG) pathway analysis for the differential genes.

**Description**	**Generatio**	**Pvalue**	**p.adjust**	**qvalue**	**geneID**
Fat digestion and absorption	6/57	4.80E-07	8.45E-05	7.84E-05	ABCG5/DGAT2/MOGAT3/APOA1/FABP1/ABCG8
Cholesterol metabolism	5/57	2.48E-05	0.002180513	0.002021408	APOA2/ABCG5/LPA/APOA1/ABCG8
PPAR signaling pathway	5/57	0.000199794	0.011721233	0.010865975	APOA2/SCD5/ACSL6/APOA1/FABP1
Protein digestion and absorption	5/57	0.000531847	0.023401248	0.02169374	COL12A1/COL3A1/SLC7A9/COL1A2/COL1A1
AGE-RAGE signaling pathway in diabetic complications	5/57	0.000672758	0.023681078	0.021953152	NOX4/AKT3/COL3A1/COL1A2/COL1A1
Central carbon metabolism in cancer	4/57	0.001465681	0.042993318	0.039856246	AKT3/KIT/PFKP/SLC2A2
Platelet activation	5/57	0.001768727	0.044470846	0.041225963	AKT3/PRKG1/COL3A1/COL1A2/COL1A1
ECM-receptor interaction	4/57	0.003392436	0.074633591	0.069187838	TNC/COL1A2/COL1A1/THBS2

*KEGG, Kyoto Encyclopedia of Genes and Genomes; PPAR, peroxisome proliferators-activated receptor; ECM, extracellular matrix. GeneRatio: Annotation of genes in this pathway, annotation of genes in all KEGG pathways, and the ratio of the two; BgRatio: All genes annotated to pathway, all genes annotated to KEGG pathways, and the ratio of the two. Pvalue: P value of hypergeometric test; P.adjust: The P value corrected by multiple hypothesis testing; Qvalue: Q value; GeneID: geneID annotated to the pathway; Count: The number of genes annotated to the pathway.

### Constructing a risk scoring model with differentially expressed genes

**Figure 5:**
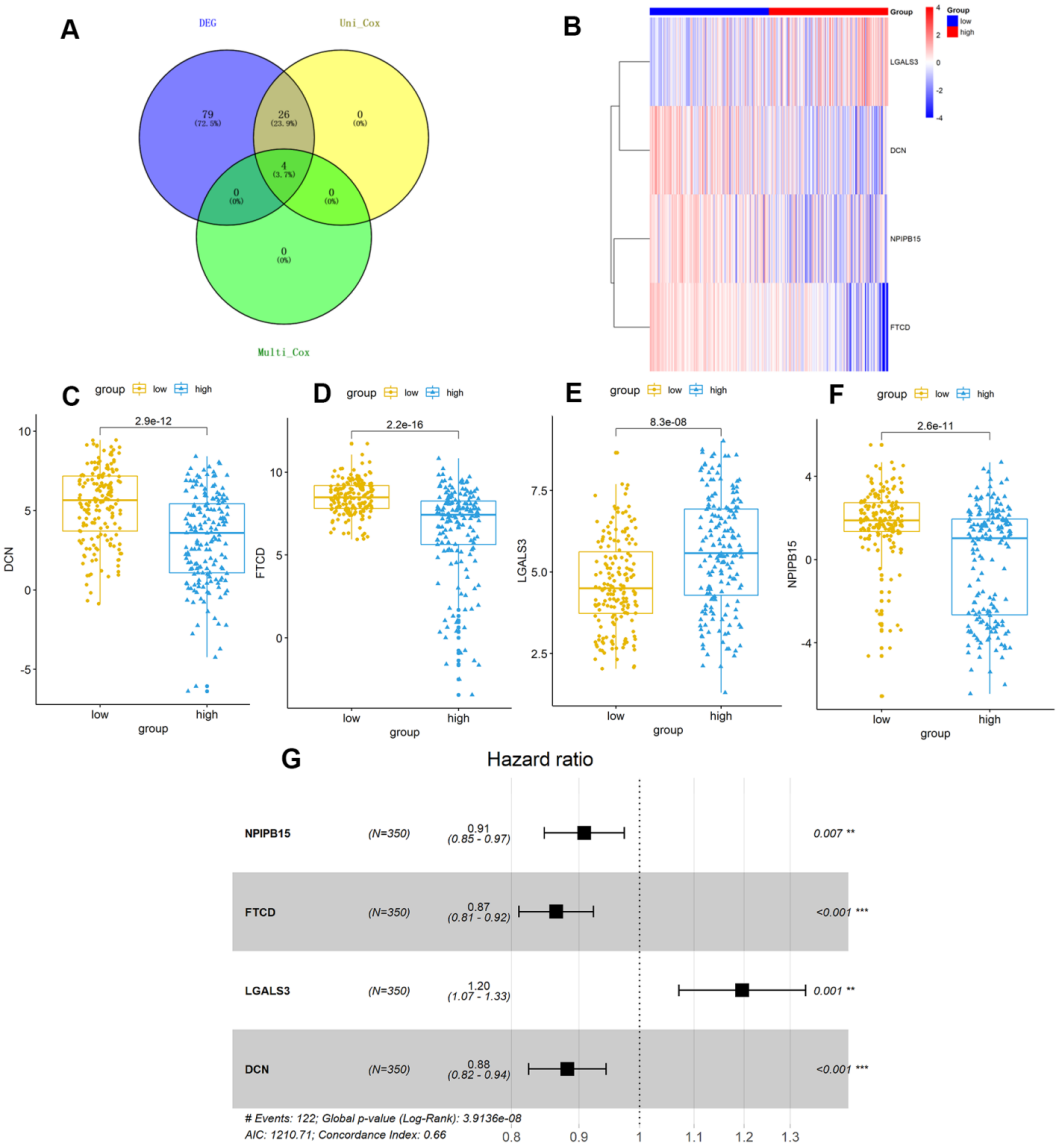
**TMB-related hub-genes analysis.** (**A**) Four TMB-related hub-genes were obtained with stepwise regression screening. (**B**) The four TMB-related hub-genes were shown in the heatmap plot. Vertical and horizontal axes represent TMB-related hub-genes and HCC samples respectively, as ranked by TMB value. Genes with higher and lower levels are shown in red and blue, respectively. Color bars at the top of the heat map represent sample types, with red and blue indicating high- and low-TMB samples, respectively. (**C**–**F**) LGALS3 (**E**) had higher expression in high-TMB group (P < 0.01), while DCN (**C**), FTCD (**D**), and NPIPB15 (**F**) were negatively correlated with high-TMB (P < 0.01). (**G**) Calculated by Cox multivariate model, hazard ratio with 95% confidence interval (95% CI) for each independent TMB-related signature are shown in forest plot. TMB, tumor mutation burden; HCC, hepatocellular carcinoma.

**Figure 6:**
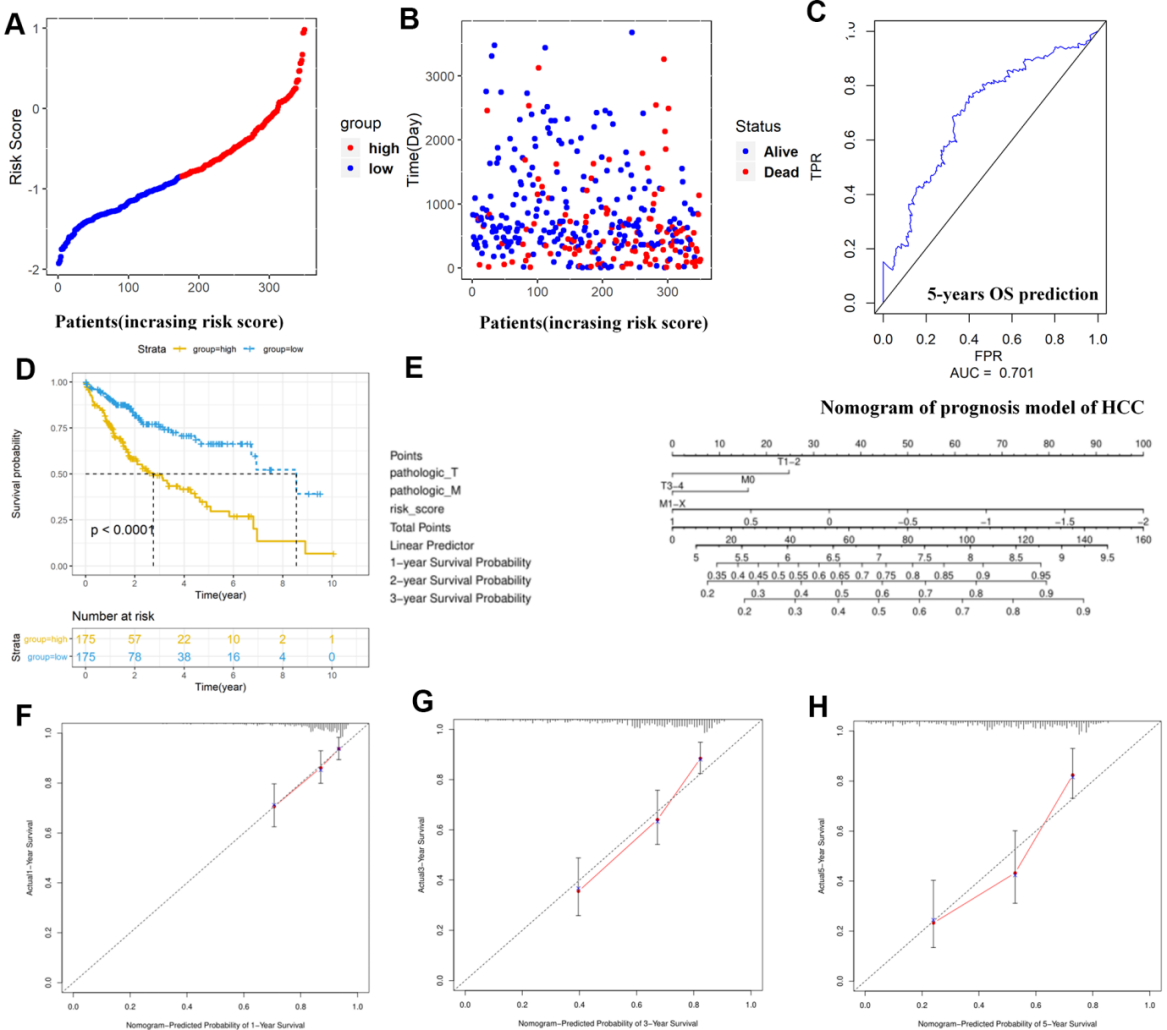
**Construction and assessment of TMBPM for HCC.** (**A**, **B**) Distribution of the risk score for each HCC patient as per the TMBPM levels. Vertical and horizontal axes respectively represent risk score and OS patients, as ranked by increasing risk score. Red and blue colors respectively represent high and low risk cases. (**B**) Dot plot of Survival time for each HCC patient as per the TMBPM levels. Vertical and horizontal axes respectively represent survival times and OS patients, as ranked by increasing risk score. Read and blue colors represent dead and living OS cases, respectively. (**C**) Horizontal and vertical axes are false positive rates and true positive rates, respectively. The AUC value of the ROC plot was 0.701 that showed superior predictive accuracy of TMBPM. (**D**) Kaplan-Meier analysis demonstrated that higher TMBPMs showed worse OS with P < 0.0001. (**E**) Nomogram of the prognosis model of HCC. (**F**–**H**) All the calibration curves of 1-year (**F**), 3-year (**G**), and 5-year (**H**) prognosis model fitted well. HCC, hepatocellular carcinoma; TMBPM, tumor mutation burden prognostic model; OS, overall survival; ROC, receiver operating characteristic; AUC, area under the curve; TPR, true positive rates; FPR, false positive rates.

Further, we identified 30 prognostic signatures associated with TMB using univariate Cox regression model from the above 109 DEGs (Supplementary Table 4). In addition, we used multivariate Cox analysis to select four independent risk signatures with p < 0.05 and acquired the coefficients (ß_i_) of the respective signature (Figure 5A and Table 3). The selection process was visualized on a Venn plot (Figure 5A). Among these four TMB-related genes, we found that the lectin galactoside-binding soluble 3 (LGALS3) expression was upregulated in high-TMB groups than in low-TMB groups, and the expression of Nuclear Pore Complex Interacting Protein Family Member B15 (NPIPB15), Formimidoyltransferase Cyclodeaminase (FTCD), and Decorin (DCN) was negatively correlated with the TMB level (Figure 5B–5F). The hazard ratio (HR) with 95% confidence interval was shown in the forest plot (Figure 5G). Using the multivariate Cox regression model, TMBPM was constructed with the following formula: TMBPM = 0.179 x LGALS3 − 0.096 x NPIPB15 − 0.145 x FTCD − 0.126 x DCN. The multivariate model indicated that a high risk score was associated with poor survival (P < 0.001) (Figure 6A, 6B, 6D). Moreover, we classified the HCC patients into high-risk (n = 175) and low-risk (n = 175) groups using the median value as the cutoff value (Figure 6C, 6D). The receiver operating characteristic (ROC) curve for 5-year OS prediction suggested that the model possessed predictive accuracy with AUC = 0.701 (Figure 6C), and Kaplan–Meier plot showed that patients with high TMBPM revealed worse OS than those with low TMBPM (Figure 6D). Furthermore, HCC prognosis model nomograms were constructed based on different clinical characteristics (Figure 6E). We calibrated the 1-year, 3-year, and 5-year OS predictions for HCC patients, and all the calibrated curves were well-fitted (Figure 6F–6H). Although TMBPM can accurately predict the 3-year and 5-year survival rate in HCC patients, whether the TMBPM maintained the independent predictive value needs to be investigated and validates on larger samples.

**Table 3:** Multivariate Cox analysis of TMB-related signature for HCC patients.

**Var**	**Coef**	**HR**	**CI95.low**	**CI95.high**	**Zscore**	**P_value**
NPIPB15	−0.096	0.908	0.847	0.974	−2.709	0.00675
FTCD	−0.145	0.865	0.811	0.923	−4.366	1.30E-05
LGALS3	0.179	1.196	1.071	1.335	3.18	0.001473
DCN	−0.126	0.882	0.824	0.943	−3.651	0.000261

*Var: Cox univariate analysis TMB-related genes; Coef: Risk coefficient; HR: relative risk value; Ci95.low, CI95.high: range of 95% confidence interval of relative risk value HR; Zscore: Z value of statistical test; P_value: the P value tested by Cox test with multi-factor; HCC: hepatocellular carcinoma; TMB: tumor mutation burden.

### Comparison of immune cell abundance between high-TMB and low-TMB groups

**Figure 7:**
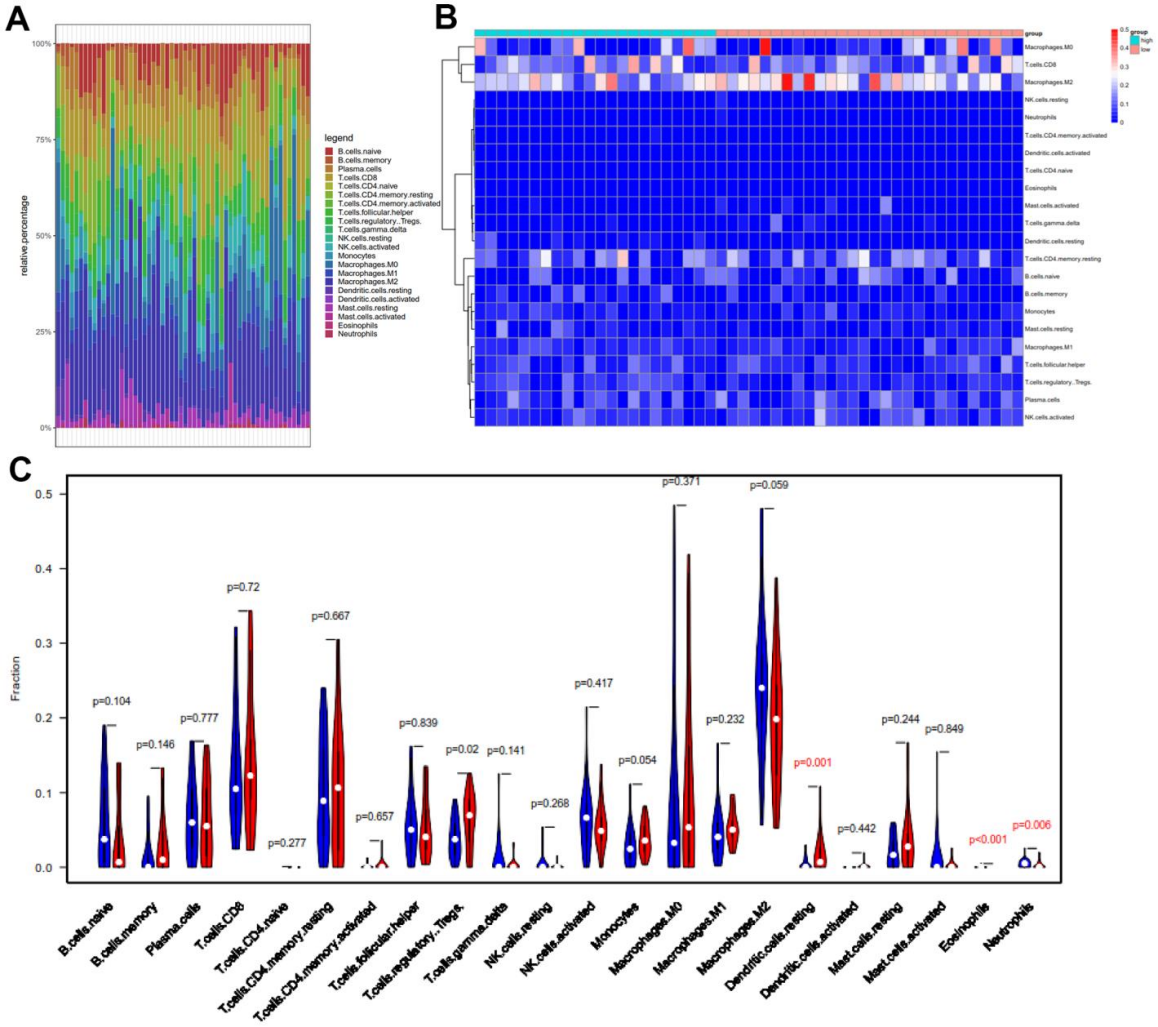
**Comparison of 22 important tumor-infiltrating immune Cells (TIICs) between the low-TMB and high-TMB groups.** (**A**) Barplot exhibited the proportion of 22 kinds of TIICs in HCC tumor samples, and the different colors represent the 22 TIICs. Vertical and horizontal axes represent relative percentage and patients, respectively. (**B**) Differential abundances of TIICs in the two groups are shown in the heatmap plot. Vertical and horizontal axes represent TIICs and patients, respectively. TIICs with higher and lower correlation levels are shown in red and blue, respectively. Color bars at the top of the heat map represent sample types, with pink and blue indicating low- and high-TMB samples, respectively. (**C**) The violin plot compared the proportions of 22 TIICS between low-TMB and high-TMB groups. Blue and red colors represent low- and high-TMB patients, respectively. Vertical and horizontal axes respectively represent TIICs fraction and TIICs, respectively. The Wilcoxon rank-sum test revealed that the infiltration levels of T cells regulatory (Tregs), dendritic cells resting and eosinophils were higher in the high-TMB group. TMB, tumor mutation burden; TIICs, tumor-infiltrating immune Cells; Tregs, T cells regulatory.

Based on the newly developed CIBERSORT software [35], we intended to compare the differential profiles of immune fractions between the high-TMB and low-TMB groups. After filtering out patients with P > 0.05 with the “CIBERSORT” package, we obtained fractions of 22 immune cells in 56 HCC patients, and the results were displayed in the box (Figure 7A), where different colors represented various cell subsets.

Meanwhile, we revealed the differential abundance of immune cells between low-TMB and high-TMB groups with heatmap plot (Figure 7B), wherein we could intuitively find that CD8+ T cell, M0 macrophage, and M2 macrophage formed the majority of the components. Moreover, the Wilcoxon rank-sum test indicated that the infiltration levels of dendritic cells resting (P = 0.001), eosinophils (p < 0.001), and T cells regulatory (Tregs) (p = 0.02) were higher in the high-TMB group than in the low-TMB group (Figure 7C). In addition, the density of neutrophils (p = 0.006) showed a lower infiltrating level in the high-TMB group. In accordance with previous mutation analysis and Kaplan-Meier analysis, lower TMB commonly inhibited the immune infiltration levels in HCC patients, contrary to clear cell renal cell carcinoma (ccRCC) [36]. Macrophages were identified using CIBERSORT in HCC. Macrophages were significantly enriched in tumors and displayed similar proportions in the immune fractions of high- and low-TMB groups. Although M2 was a dominant Macophages, there were not clearly distinguished in M0, M1 and M2 macrophages, consistent with other previous reports [37, 38].

**Figure 8:**
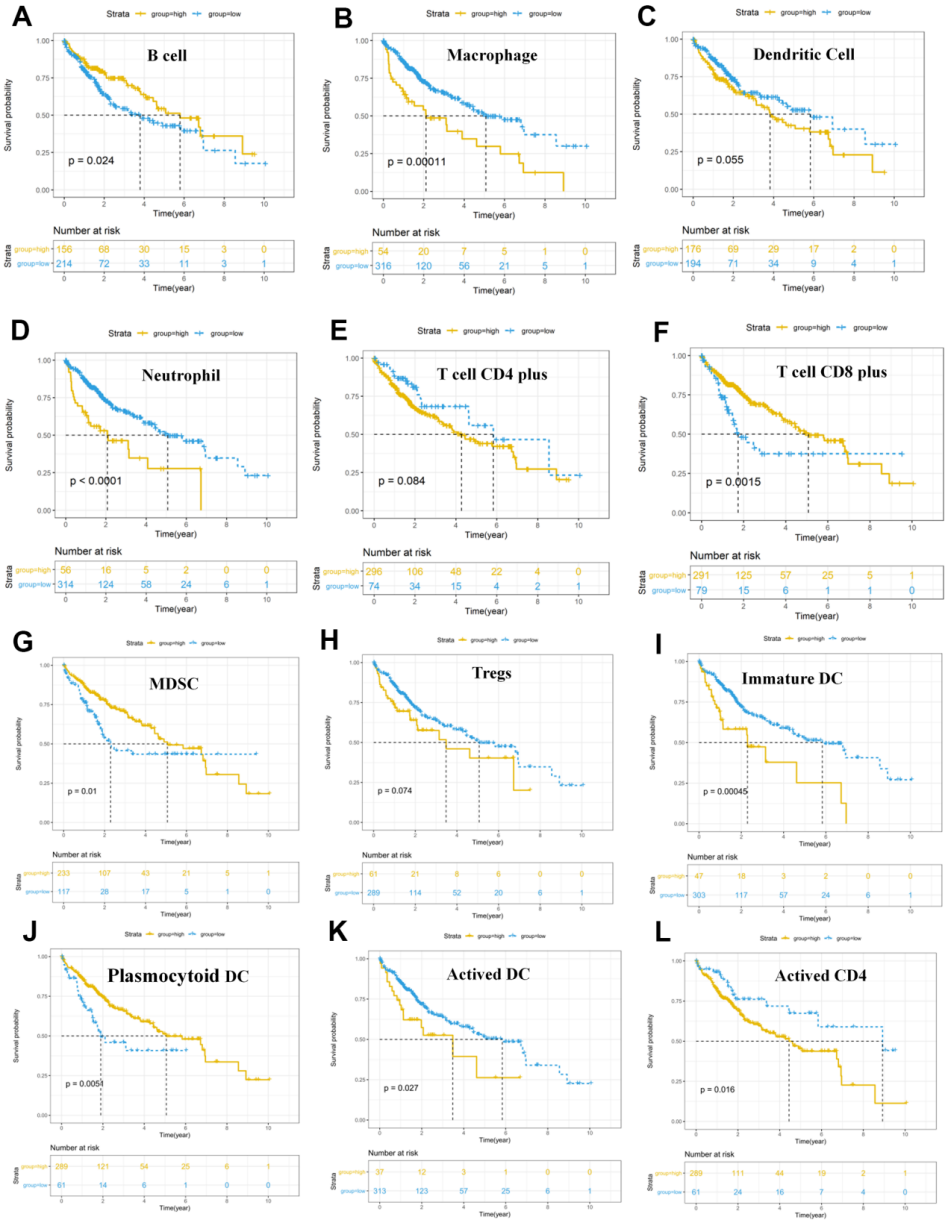
**Survival analysis of immune cells based on the TIMER database.** Horizontal and vertical axes represent survival times and survival rates, respectively. Yellow and blue curves are samples with higher and lower immune cell fractions, respectively. (**A**–**L**) Lower infiltration levels of macrophages (**B**), dendritic cells (**C**), and neutrophil (**D**), CD4+ T cells (**E**), Tregs (**H**), Immature DC (**I**), Activated DC (**K**) and Activated CD4 (**L**) with improved survival outcomes, and higher infiltration levels of B cells (**A**), CD8+ T cells (**F**), MDSC (**G**) and Plasmocytoid DC (**J**) were associated with poor survival outcomes in HCC. HCC, hepatocellular carcinoma; TIMER, Tumor Immune Estimation Resource; Tregs, T cells regulatory; MDSC, myeloid-derived suppressor cells.

We intended to compare the differential profiles of immune fractions between the high TMB and low TMB groups; therefore, we further intended to investigate the potential prognosis of immune cells based on the Tumor Immune Estimation Resource (TIMER) database. Multivariate Cox analysis showed that higher macrophage (HR = 29.333, P = 0.023), dendritic cells (HR = 21.823, P = 0.013) infiltrates comprised hazard factors with poor OS rates (Table 4 and Figure 8B, 8C), while the density of B cells (HR = 0.005, P = 0.040) or CD8+ T cell (HR = 0.021, P = 0.006) were marginally protective infiltrating cells (Table 4). Furthermore, the Kaplan–Meier analysis explained that higher infiltration levels of CD8+ T cells and B cells were associated with improved survival outcomes in HCC (Figure 8C).

**Table 4:** Multivariate Cox regression analysis of the immune infiltration cells based on TIMER database in HCC.

**Tumor-infiltrating immune cell**	**Coef**	**HR**	**CI95.low**	**CI95.high**	**Zscore**	**P_value**
B_cell	–5.334	0.005	0.000	0.787	–2.052	0.040
T_cell_CD4_plus	–3.77	0.023	0.000	2.740	–1.547	0.122
T_cell_CD8_plus	–3.879	0.021	0.001	0.343	–2.705	0.007
Neutrophil	3.561	35.203	0.078	15938.055	1.141	0.254
Macrophage	3.379	29.333	1.567	549.224	2.260	0.024
Dendritic_cell	3.083	21.823	1.927	247.152	2.490	0.013

*Var: Cox univariate analysis immune cells; Coef: Risk coefficient; HR: relative risk value; Ci95.low, CI95.high: range of 95% confidence interval of relative risk value HR; Zscore: Z value of statistical test; P_value: the P value tested by Cox test with one factor. HCC: hepatocellular carcinoma.

**Figure 9:**
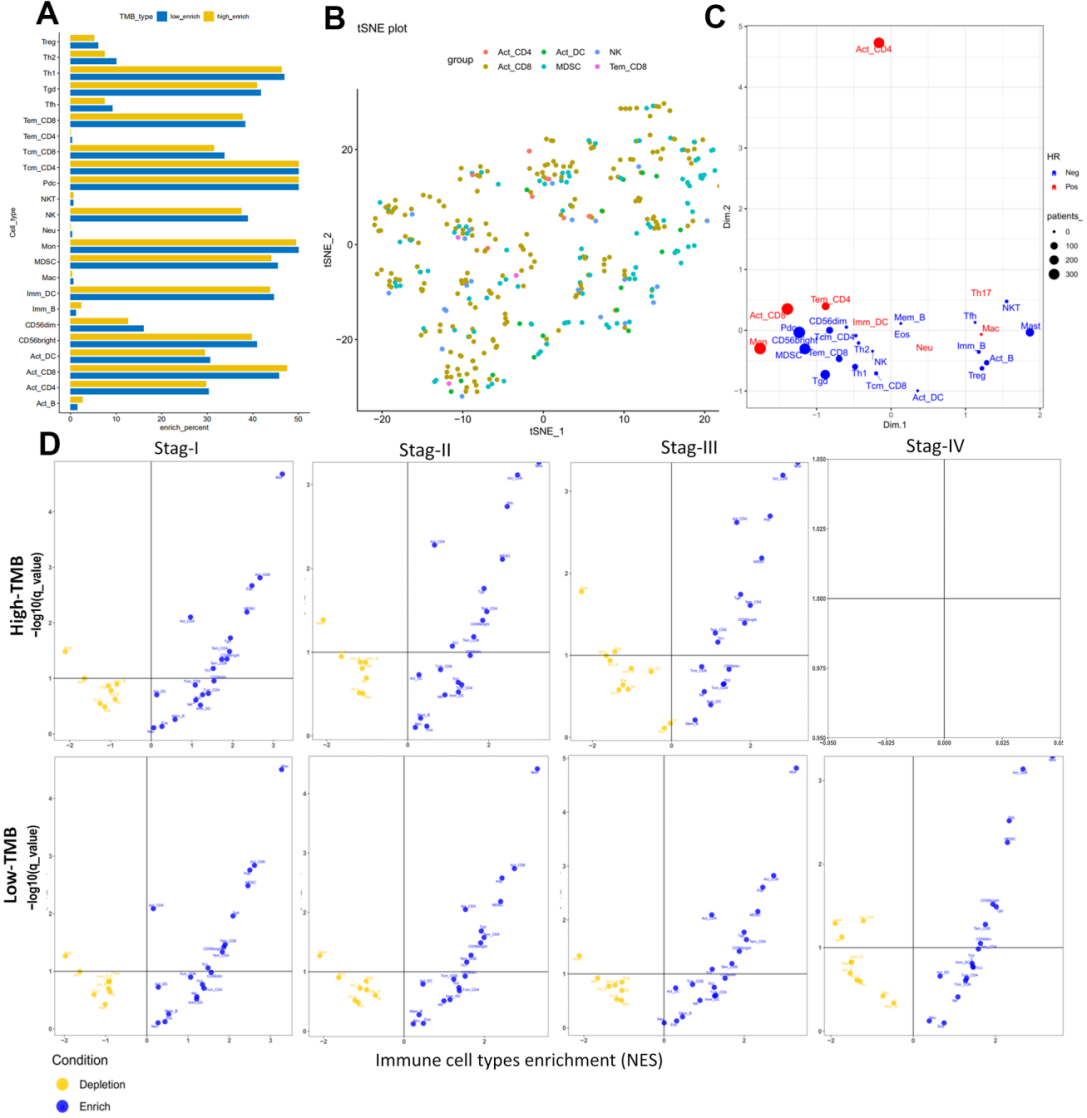
**Cellular characterization of immune infiltrates based on GSEA database.** (**A**) Correspondence analysis of immune subpopulations in HCC. Horizontal and vertical axes represent enrich percent and immune subpopulations, respectively. Yellow and blue bar are samples with higher and lower immune cell fractions, respectively. (**B**) t-SNE showing individual patients and selected cell types based on two dimensional coordinates. Different colors are different immune subpopulations respectively. (**C**) Visualization of the immune infiltrates for all patients using two dimensional coordinates from multidimensional scaling (MDS). (**D**) Volcano plots for the enrichment (blue) and depletion (yellow) of immune cell types across cancers for tumor stage I to IV calculated based on the NES score in high-TMB (up panel) and low-TMB (down panel) from the GSEA. Horizontal and vertical axes represent Immune cell types enrichment (NES) and −log10(q−value), respectively. There were not any pots in stage IV for high-TMB, because there were not enough samples for analysis.

Furthermore we investigate 28 subpopulations of immune cells based on the GSEA database (https://tcia.at/home) (Figure 9A and Table 5). The identified immune cells included T cells (activated T cells, central memory (Tcm), effector memory (Tem) CD4+ and CD8+ T cells, gamma delta T cells (Tγδ), regulatory T cells (Treg), follicular helper T cells (Tfh), T helper 1 (Th1), Th2, Th17), B cells (activated, immature, and memory B cells), myeloid-derived cells (macrophages, activated, plasmocytoid and immature dendritic cells (DCs), monocytes, mast cells, eosinophils, neutrophils,), NK, natural killer T cells (NKT), and MDSCs. All these cell subtypes were shared between the two-groups, albeit at different proportions (Figures 7C, 9A). The infiltration levels of B cells, tregs were relatively low in all patients, meanwhile the infiltration levels of DCs and the other T cells were higher in all patients (Figures 7C, 9A) consistent with a previous report [37]. Other immune cell types varied between high TMB and low TMB patients, revealing substantial heterogeneity of immune cell compositions among HCC tumors (Figures 7C, 9A).

**Table 5:** Univariate regression analyses of immune infiltration cells based on GSEA database in HCC.

**Tumor-infiltrating immune cell**	**All samples**			**High-TMB**			**low-TMB**	
	**HR**	**P.value**		**HR**	**P.value**		**HR**	**P.value**
Act_CD8	2.415	0.002		0.559	0.160		0.621	0.129
Tcm_CD8	0.881	0.085		11.825	0.045		1.138	0.897
Tem_CD8	0.754	0.013		1.465	0.646		0.605	0.478
Act_CD4	1.120	0.008		3.003	0.359		0.864	0.844
Tcm_CD4	0.342	0.011		1.097	0.859		0.774	0.508
Tem_CD4	2.612	0.004		NA	NA		NA	NA
Tfh	0.982	0.842		0.137	0.542		16.591	0.153
Tgd	0.849	0.059		5.022	0.132		1.194	0.818
Th1	0.868	0.073		0.433	0.280		0.623	0.377
Th17	1.015	0.943		NA	NA		NA	NA
Th2	0.911	0.473		0.921	0.984		3.191	0.427
Treg	0.966	0.647		NA	NA		2.241	0.557
Act_B	0.821	0.027		NA	NA		NA	NA
Imm_B	0.903	0.215		NA	NA		NA	NA
Mem_B	0.814	0.044		NA	NA		NA	NA
NK	0.732	0.008		0.527	0.503		0.837	0.784
CD56bright	0.621	0.242		1.012	0.992		1.198	0.839
CD56dim	0.742	0.582		7353.518	0.060		0.471	0.739
MDSC	0.730	0.307		0.942	0.893		1.176	0.668
NKT	0.883	0.720		NA	NA		NA	NA
Act_DC	0.982	0.780		1.291	0.779		2.251	0.210
Pdc	0.576	0.250		1.606	0.428		0.302	0.021
Imm_DC	1.241	0.417		10.151	0.022		2.937	0.151
Mac	1.040	0.778		NA	NA		NA	NA
Eos	0.937	0.597		NA	NA		NA	NA
Mast	0.612	0.032		NA	NA		NA	NA
Mon	1.169	0.635		0.556	0.454		0.836	0.763
Neu	1.029	0.814		NA	NA		NA	NA

HR: relative risk value; P_value: the P value tested by Cox test with multi-factor; NA: The sample size is too small for Cox analysis; HCC: hepatocellular carcinoma, TMB, tumor mutation burden.

### DCs infiltrates in HCC indicated survival outcomes

The results of the cellular characterization of the immune infiltrates using GSEA showed that the infiltration of Tem CD8+ cells (HR = 0.754, p = 0.013), Tcm CD4+ cells (HR = 0.342, p = 0.011), Activated B cell (HR =0.821, p = 0.027), Memory B cell (HR =0.814, p = 0.044), Natural killer (NK) cell (HR = 0.732, p = 0.008) and Mast cell (HR = 0.612, p = 0.032) were associated with good prognosis, whereas activated CD8+ cells (HR = 2.415, p = 0.002), activated CD4+ cells (HR = 1.120, p = 0.008) and Tem CD4+ cells (HR = 2.612, p = 0.004) were associated with bad prognosis (Table 5). Although higher myeloid-derived suppressor cells (MDSCs) infiltration and lower Tregs infiltration associated with improved OS (Figure 8G, 8H), MDSCs (HR = 0.730, p = 0.307) and Tregs (HR = 0.966, p = 0.647) (Table 5) which are significantly associated with bad prognosis in other cancer [39] were not hazard risk (Table 5) for HCC in our study, and we also don’t observed separation of the MDSCs and Tregs subpopulations related to immune suppression from the subpopulations related to the effector function (activated T cells, Tcm, Tem CD4+ and CD8+ cells) (Figure 9B, 9C). The separation was found in other cancers including lung squamous cell carcinoma, ovarian cancer, pancreatic cancer, and melanoma [39].

The progression was also characterized by distinct immune cell patterns between high- and low-TMB group based on GSEA database. Within each TMB group, the composition of the immune cells was divergent in HCC. For example, activated DCs, EOS and Neu were enriched in stage I and stage II tumors and only depleted in stage III in high-TMB patients (Figure 9D), and that activated CD4 and Mem_B were only depleted in late stage tumors and enriched in early stage tumors (Figure 9D) in low-TMB samples. There were not any pots in stage IV for high-TMB, because there were not enough samples for analysis (Figure 9D). Meanwhile, Univariate Cox analysis showed that higher levels of mDCs (HR = 21.823, P = 0.013) and Imm_DC (HR = 10.151, p = 0.022) with poor OS rates (Tables 4, 5 and Figure 8C, 8I) were hazard factors in High TMB group, while higher density of pDCs (HR = 0.302, p = 0.021) with improved OS rates (Figure 8J, P < 0.01) was marginally protective infiltrating cells in low-TMB groups. These results might explain why some patients with high TMB are not responsive to therapy with checkpoint blockers and also why some patients with low TMB are responders.

In summary, these analyses showed that both the genomic profiles and the specific tissue context contribute to the cellular composition of the immune infiltrates. Furthermore, whether using CIBERSORT, TIMER database or GSEA database, it is noteworthy that inflated levels of DCs and prognosis of DCs were different between high-TMB and low-TMB groups. mDCs and immature DCs subsets showing higher infiltrating abundance in the high TMB group was associated with lowered OS and higher risk factor, indicating that these high TMB HCC patients with higher infiltrates of mDCs and immature DCs had poor survival outcomes, meanwhile the higher fraction of pDCs was lower risk factor and was associated with better OS in low TMB samples. Accordingly, Kaplan–Meier analysis and Wilcoxon rank-sum test showed that dendritic cells can also be used as secondary prognosis indicators for immunotherapy in HCC.

## DISCUSSION

Liver cancer (LC) remains the most common cause of cancer-related death and the second major cause of cancer-related death worldwide, despite considerable improvements in its treatment [2]. Since 2017, immune checkpoint inhibitors (ICIs) have showed promising results in the immunotherapy for advanced HCC [9]. However, few HCC patients are able to obtain benefits from this treatment. Thus, many researches have focused on the identification of predictive biomarkers for immunotherapy.

In the current research, we found that mutations in TP53 (28%), CTNNB1 (24%), and TTN (25%) are frequently found in HCC (Figure 1). Wang found that HCC patients with TP53 mutation were significantly correlated with high TMB (P = 0.0005) and exhibited poor prognosis (OS: HR = 1.58, P = 0.0109) [40], indicating that HCC patients with TP53 mutation were more likely to benefit from immune treatment. Furthermore, CTNNB1 mutation was positively correlated with TMB-H and TP53 in HCC [41]. A meta-analysis found that NSCLC patients with EGFR mutation could not benefit from immunotherapy, and EGFR-positive patients had low TMB [42, 43].

TMB is a novel prognosis biomarker for ICIs therapy in breast cancer [44] and other tumors [45, 46]. Robert found that high somatic TMB patients treated with ICIs exhibited better OS [45]. Hellmann demonstrated that small-cell lung cancer patients with high TMB treated with either nivolumab plus ipilimumab or nivolumab monotherapy had better prognosis, irrespective of the level of PD-L1 expression [25]. In this study (Figure 2), HCC patients with high TMB had significantly better OS (p = 0.0014) than those with other malignancies [33]; moreover, higher TMB may induce immune recognition and prognosis improvement. Further, TMB was negatively correlated with the tumor stages (P = 0.035) and AJCC-T stages (P = 0.027) (Figure 2E). These results suggest that the TMB level declined with tumor invasion and progression; this prompted us to investigate the potential relationship among TMB, DEGs, and immune infiltrates for identifying more prognostic biomarkers for HCC.

Subsequently, four hub TMB-related signatures were identified (Figure 5 and Table 3) on univariate and multivariate Cox analysis (positive correlation: LGALS3, negative correlation: NPIPB15, FTCD and DCN). Furthermore, a prognostic model (TMBPM) was developed using four hub TMB-related signatures that can be very useful for survival prediction (Figure 6 and Table 3). To our knowledge, this is the first TMB prognostic model to predict survival outcomes in HCC.

Patients with a high TMBPM score had worse survival outcomes. Moreover, the AUC of the ROC curve was 0.701, indicating the prediction accuracy of the TMBPM. Although we calibrated the curves of 1-year, 3-year, and 5-year prognosis prediction models, further research on larger samples is needed before clinical application. In bladder cancer (BLCA) [33], tumor mutation burden related signature (TMBRS) mode were constructed by eight hub TMB-related signatures for prognosis prediction. Although this method can accurately predict the 3-year and 5-year survival rate in HCC and BLCA, whether the method maintained the independent predictive value needs to be investigated and validates on larger samples in different cancers.

LGALS3 (Galectin-3) is mainly involved in cell growth, cell adhesion, cell differentiation, and tumor progression and metastasis owing to its action of binding to glycoproteins. Kada found that decreased expression of Galectin-3 in gastric cancer indicated poor prognosis [47]. The core proteoglycan (DCN), a main component of ECM, negatively regulates Tregs [48–52]. DCN can indirectly inhibit the formation of foxp3+ Tregs via the inhibition of the TGF-β signaling pathway [53, 54]. Many studies have demonstrated that DCN overexpression inhibits the progress of various tumors, such as breast cancer and colon cancer [35, 55, 56].

Low expression of FTCD is correlated with poor prognosis (P < 0.001) in HCC as per the TCGA data [57]; meanwhile, FTCD overexpression suppressed the proliferation of BEL-7402 and SNU499 cells, resulting in increased PTEN protein and decreased PI3K, total Akt, and phosphorylated Akt protein in BEL-7402 and SNU499 cells. As per a recent study, the PTEN-PI3K/AKT signal transduction pathway was involved in tumor immune escape via the regulation of PD-L1 expression [58]. PD-L1 played an important role in inducing specific T cell apoptosis and tumor immune escape. Thus, FTCD overexpression inhibits tumor progression and tumor immune escape in HCC via the suppression of PI3K/AKT pathway activation. Therefore, FTCD could also be a promising biomarker and a potential target for HCC treatment.

TIMER is the first method for performing integrative analysis of tumor immune cell, clinical, and genomics data [59]. TIMER database was used for assessing the relationships of 9 TMB-related signature with immune infiltration levels in clear cell renal cell carcinoma (ccRCC) [36], the 9 signatures were associated with lower immune infiltrates. After constructing the TMB prognostic model, we also compared the abundance of immune cells utilizing TIMER database, CIBERSORT software, and GSEA database, and found that the prognosis of DCs infiltration was different between high- and low-TMB group. At present, DCs are considered as prognostic indicators in cancers, because the higher infiltrated DCs are associated with better prognosis [60, 61]. Cai et al. found that high infiltration of DC in hepatoma indicates a higher disease-free survival time [60]. Single-cell sequencing of 16,498 HCC cells found that, compared with primary hepatocellular carcinoma, more DCs and CD8+ T cells were infiltrated in early recurrent tumors, while fewer regulatory T cells played an immunosuppressive role [37] in early recurrent tumors. Due to the high affinity of PD-L1 binding to CD80 on the DC surface, CD80 was preferentially bound to PD-L1. Thereby the competitive inhibition that CD80-CD28 mediated CO-stimulation of DC on CD8+ T cells blocked antigen presentation and inhibited the activation of CD8+ T cells. This suggests that the mechanism of immune escape in early recurrent tumors is different from that in primary hepatocellular carcinoma. This will contribute to the development of more effective therapeutic targets and biomarkers for immunotherapy in HCC patients [37]. According to the origin, DCs are divided into DCs derived from myeloid DC (mDC) and lymphatic dendritic cells (LDC) or plasmacytoid dendritic cells (pDC) [62]. In this study, the prognosis of different DC subgroups was different between high-TMB and low-TMB groups. For example, higher fraction of pDCs associated with improved OS rates (Table 5 and Figure 8J) was marginally protective infiltrating cells and immune response in low-TMB group, while higher fraction of pDCs was a hazard risk for high-TMB patients (Table 5). Previous research results shown that Mature pDCs inhibited the tumor, while immature pDCs in the tumor microenvironment promoted the tumor [63, 64]. These previous research results might explain that higher infiltration of mature pDCs was favorable factor while immature pDCs was risk factor in HCC. On the other hand, although the fraction of pDCs was not different between the two TMB groups (Figure 9A), pDCs is might mainly mature pDCS in low TMB while immature pDCS in high TMB in HCC. However, this hypothesis needs to be verified by flow cytometry, and/or immunohistochemistry.

In this study, we found that high-TMB patients had higher infiltration levels of resting dendritic cells (DCs) (Figure 7), poor OS (Figure 8), and that higher infiltration levels of mDCs (Table 4), especially the immature DCs (Table 5) was a hazard factor in HCC based on TIMER database and GSEA database. Myeloid-DCs (mDCs) can induce both primary immunosuppression and tumorigenesis [65]. mDCs are considered to be a negative factor in anti-tumor immunity due to their decreased expression and function [66]. Further, in the present study, the infiltration level density of T cells regulatory (Tregs) was higher in the high-TMB group. Previous studies have shown that Tregs could inhibit the proliferation of CD4+ T cells and the secretion of IL-2 [67, 68]. SATO found that the improvement in tumor-specific CD8+ T cells and reduction in Tregs cells can effectively improve the prognosis in cancer patients [69]. Empirical researches have shown that mDCs are related to the selection of thymic Tregs, differentiation, proliferation, and functional regulation of peripheral lymphoid tissues [70, 71].

Although higher macrophage level was a hazard risk factor associated with poor survival outcomes, which is in agreement with that of breast cancer [72], no significantly different macrophage levels were observed in the two TMB groups. Higher MDSCs infiltrations was associated with improved OS, but we couldn’t cleanly distinguish the difference of MDSCs infiltrations levels and risk factor of MDSCs between high- and low-TMB groups in HCC. Therefore, we could not easily determine the potential relationship between TMB, infiltration of macrophage and MDSCs.

In our study, infiltrating levels of DCs, TMB, and TMB-related hub signatures can be used as prognostic indicator in HCC. However, TMB should be analyzed together with DCs infiltrating levels, especially in stage III and stage IV patients, wherein TMB is generally lower. Moreover, the four TMB-related signatures can also be prognosis index in HCC. While there are some limitations should be taken into consideration: (1) although the role of TMB in the prognosis of HCC and its potential relationship with immune cell infiltration had been identified by CIBERSORT software, TIMER database and ssGSEA, different analysis methods also have conflicting conclusions, which need to be verified through a large number of clinical data or clinical trials in the future; (2) The four TMB-related genes and immune cell infiltrates are also needed to be validated based on basic experiment in the future.

## CONCLUSIONS

In conclusion, higher TMB correlated with improved survival outcomes and might induce local immune recognition in HCC. Patients were divided in two risk groups using TMBPM based on 4 hub TMB-related signature, and TMBPM index may serve as a promising prognostic biomarker for HCC in the future. Different DC subgroups (mDCS, pDCs and immDCs) and the infiltrating levels of these DC subgroups were different risk factors and also were associated with different survival outcomes between high- and low-TMB samples, while may play an important role in metastasis, suppression and progress of HCC.

## MATERIALS AND METHODS

### Acquisition of multi-omics data resource

All the data used in this article were obtained from the TCGA database on the GDC website (https://portal.gdc.cancer.gov/), including HCC mutation data; transcriptome profiles; and clinical information, such as age, sex, AJCC-TNM stages, pathological stages, tumor stages, and survival outcomes. The statistical results of somatic mutation were visualized with the maftools software. The transcriptome profiles with HTSeq-FPKM Format of 371 patients were also downloaded from the TCGA database with the GDC tool.

### Correlation analysis between TMB and prognosis

First, we filtered out the germline mutations annotated in the dbsnp and ExAC databases. Then, we defined and calculated the TMB of each sample as the total amount of coding variants/the length of exons (38 million), where the detected variants were considered as frameshift deletion mutation, in-frame deletion, frameshift insertion mutation, in-frame insertion, missense mutation, nonsense mutation, nonstop mutation and silent. As per the median TMB value, the patients were divided into the high-TMB and low-TMB groups. Kaplan–Meier survival analysis with log-rank test was performed to assess the differential survival rate between the high-TMB and low-TMB groups. In order to improve the accuracy of Kaplan–Meier survival analysis, we excluded these samples with survival duration of <10 d. Moreover, the association of TMB distribution with several clinical variables (AJCC-TNM stage, tumor stage, and case stage) was evaluated using Kruskal–Wallis test.

### Differential expressed genes and functional analysis in the 2 TMB groups

For the high-TMB and low-TMB groups, we used the limma software to identify DEGs in the two groups with |logFC| > 1.5 and FDR < 0.05. The heatmap was displayed with the "heatmap" package to visualize the differences. ClusterProfiler package was implemented for GO and KEGG analysis with q value < 0.05. Furthermore, GSEA analysis was performed with FDR < 0.3 based on JAVA8 platform using the TMB level as the phenotype. Moreover, STRINGdb software was used for protein–protein interaction analysis.

### Identification of prognostic genes

Thirty TMB-related genes were obtained from 109 DEGs using univariate Cox survival analysis with P < 0.05. Subsequently, four hub TMB-related signatures were identified using the stepwise regression screening method; the process has been shown in the form of a Venn diagram. TMBPM was used to calculate TMBPM = Ʃ (βi × EXPi) based on multivariate Cox analysis. ROC curve was utilized to assess the predictive score of four TMB-related signatures in HCC.

The nomogram of the HCC prognosis model was established via univariate and multivariate analyses, combined with clinical characteristics. Then, calibration curves were constructed for the prediction of 1-year, 3-year, and 5-year survival in HCC.

### Assessment of immune-infiltrating cells and prognostic analysis

CIBERSORT software (https://cibersort.stanford.edu) was used to evaluate the compositions of the immune cells in HCC samples, with P > 0.05 to improve accuracy of the estimated results, Heatmap has been used to show the distribution of immune cell fractions, and violin plot visualizes the differential distributions of cells with two TMB levels.

Based on the TIMER database, multivariate Cox analysis that was fitted by function coxph (β_i_) from R package “survival” was used to identify the immune infiltration cells. Subsequently, we calculated the HR with 95% confidence interval (95%CI). Further, Kaplan–Meier survival analysis was performed with a P value < 0.05 of log-rank test to show the differential survival outcomes between different levels of immune infiltrates in HCC.

Subsequently, we identified 28 kinds of immune cells that are over-represented in the tumor microenvironment using single sample gene set enrichment analysis (ssGSEA) [39]. All immune cell types were considered enriched in a patient or group of patients when FDR (q-value) ≤ 0.1 and normalized enrichment score (NES) >0. The similarity of the enrichment of immune infiltrates (averaged NES) were calculated using multidimensional scaling (MDS). The distribution of selected cell types for individual patients were analyzed with t-distributed stochastic neighbor embedding (t-SNE) using the Matlab toolbox t-SNE.

### Statistical analyses

All the statistical analyses were conducted using R software (Version 3.5.2). The p-values were adjusted for multiple testing based on FDR according to the Benjamini-Hochberg approach, P value < 0.05 was regarded statistically significant. Differential analysis and normalization were mainly conducted by “limma” package of the R software (version 3.5.2). The Kaplan–Meier analysis with log-rank test or Cox regression model was performed by “survival” package. Student’s t test was used for continuous variables, while categorical variables were compared by χ2 test. The non-parametric two-sided Wilcoxon-rank sum test was utilized for comparing two groups and the Kruskal–Wallis test was suitable when it comes to two or more groups. Differences and correlations among immune cells were analyzed with the vioplot (https://cran.r-project.org/web/packages/vioplot/index.html).

## Supplementary Material

Supplementary Table 1

Supplementary Table 2

Supplementary Table 3

Supplementary Table 4
