# Supplementary material for: Prognostic analysis of tumor mutation burden and immune infiltration in hepatocellular carcinoma based on TCGA data
Source: Aging (Albany NY). 2021 Apr 4;13(8):11257–80. doi: 10.18632/aging.202811 (PMC8109113; doi:10.18632/aging.202811)
Supplement: Supplementary Table 1 [file aging-13-202811-s001.doc]

**Supplementary Table 1. Calculation of TMB for 361 HCC patients.**

| **Sample** | **TMB** |
| --- | --- |
| TCGA-2V-A95S | 2.368421053 |
| TCGA-2Y-A9GS | 1.184210526 |
| TCGA-2Y-A9GT | 2.131578947 |
| TCGA-2Y-A9GU | 3.157894737 |
| TCGA-2Y-A9GV | 1.657894737 |
| TCGA-2Y-A9GW | 1.842105263 |
| TCGA-2Y-A9GX | 1.394736842 |
| TCGA-2Y-A9GY | 1.631578947 |
| TCGA-2Y-A9GZ | 2.342105263 |
| TCGA-2Y-A9H0 | 1.894736842 |
| TCGA-2Y-A9H1 | 3.105263158 |
| TCGA-2Y-A9H2 | 1.078947368 |
| TCGA-2Y-A9H3 | 4.105263158 |
| TCGA-2Y-A9H4 | 1.526315789 |
| TCGA-2Y-A9H5 | 1.736842105 |
| TCGA-2Y-A9H6 | 1.421052632 |
| TCGA-2Y-A9H7 | 1.631578947 |
| TCGA-2Y-A9H8 | 1.5 |
| TCGA-2Y-A9H9 | 2.842105263 |
| TCGA-2Y-A9HA | 3.131578947 |
| TCGA-2Y-A9HB | 1.789473684 |
| TCGA-3K-AAZ8 | 3.447368421 |
| TCGA-4R-AA8I | 20.89473684 |
| TCGA-5C-A9VG | 3.157894737 |
| TCGA-5C-A9VH | 2.289473684 |
| TCGA-5C-AAPD | 1.684210526 |
| TCGA-5R-AA1C | 2.394736842 |
| TCGA-5R-AA1D | 0.421052632 |
| TCGA-5R-AAAM | 1.947368421 |
| TCGA-BC-4073 | 0.394736842 |
| TCGA-BC-A10Q | 0.131578947 |
| TCGA-BC-A10R | 0.342105263 |
| TCGA-BC-A10T | 0.184210526 |
| TCGA-BC-A10U | 0.421052632 |
| TCGA-BC-A10W | 0.473684211 |
| TCGA-BC-A10X | 0.052631579 |
| TCGA-BC-A10Y | 0.210526316 |
| TCGA-BC-A10Z | 0.921052632 |
| TCGA-BC-A216 | 0.210526316 |
| TCGA-BC-A217 | 0.342105263 |
| TCGA-BC-A3KF | 0.315789474 |
| TCGA-BC-A5W4 | 0.131578947 |
| TCGA-BC-A69H | 0.210526316 |
| TCGA-BC-A8YO | 1.631578947 |
| TCGA-BD-A2L6 | 1.947368421 |
| TCGA-BD-A3EP | 0.342105263 |
| TCGA-BD-A3ER | 1.263157895 |
| TCGA-BW-A5NO | 0.394736842 |
| TCGA-BW-A5NP | 0.157894737 |
| TCGA-BW-A5NQ | 0.421052632 |
| TCGA-CC-5258 | 0.473684211 |
| TCGA-CC-5259 | 0.368421053 |
| TCGA-CC-5260 | 0.289473684 |
| TCGA-CC-5262 | 0.421052632 |
| TCGA-CC-5263 | 0.5 |
| TCGA-CC-5264 | 0.473684211 |
| TCGA-CC-A123 | 0.263157895 |
| TCGA-CC-A1HT | 0.184210526 |
| TCGA-CC-A3M9 | 0.394736842 |
| TCGA-CC-A3MA | 0.131578947 |
| TCGA-CC-A3MB | 0.210526316 |
| TCGA-CC-A3MC | 0.368421053 |
| TCGA-CC-A5UC | 0.184210526 |
| TCGA-CC-A5UD | 4.815789474 |
| TCGA-CC-A5UE | 0.289473684 |
| TCGA-CC-A7IE | 3.868421053 |
| TCGA-CC-A7IF | 0.631578947 |
| TCGA-CC-A7IG | 0.5 |
| TCGA-CC-A7IH | 1.289473684 |
| TCGA-CC-A7II | 0.526315789 |
| TCGA-CC-A7IJ | 0.5 |
| TCGA-CC-A7IK | 0.552631579 |
| TCGA-CC-A7IL | 0.447368421 |
| TCGA-CC-A8HS | 1.263157895 |
| TCGA-CC-A8HT | 4.894736842 |
| TCGA-CC-A8HU | 2.236842105 |
| TCGA-CC-A8HV | 3.236842105 |
| TCGA-CC-A9FS | 2.657894737 |
| TCGA-CC-A9FU | 1.078947368 |
| TCGA-CC-A9FV | 0.394736842 |
| TCGA-CC-A9FW | 3.394736842 |
| TCGA-DD-A113 | 0.421052632 |
| TCGA-DD-A114 | 0.5 |
| TCGA-DD-A115 | 0.289473684 |
| TCGA-DD-A116 | 0.421052632 |
| TCGA-DD-A118 | 0.368421053 |
| TCGA-DD-A119 | 0.184210526 |
| TCGA-DD-A11A | 0.368421053 |
| TCGA-DD-A11B | 0.078947368 |
| TCGA-DD-A11C | 0.236842105 |
| TCGA-DD-A11D | 0.473684211 |
| TCGA-DD-A1E9 | 0.078947368 |
| TCGA-DD-A1EA | 0.078947368 |
| TCGA-DD-A1EB | 0.526315789 |
| TCGA-DD-A1EC | 0.105263158 |
| TCGA-DD-A1ED | 0.052631579 |
| TCGA-DD-A1EE | 4.842105263 |
| TCGA-DD-A1EF | 0.394736842 |
| TCGA-DD-A1EH | 0.157894737 |
| TCGA-DD-A1EI | 0.157894737 |
| TCGA-DD-A1EJ | 0.210526316 |
| TCGA-DD-A1EK | 0.026315789 |
| TCGA-DD-A1EL | 0.263157895 |
| TCGA-DD-A39V | 0.184210526 |
| TCGA-DD-A39W | 0.815789474 |
| TCGA-DD-A39X | 0.315789474 |
| TCGA-DD-A39Z | 0.210526316 |
| TCGA-DD-A3A2 | 0.236842105 |
| TCGA-DD-A3A3 | 0.236842105 |
| TCGA-DD-A3A4 | 0.052631579 |
| TCGA-DD-A3A5 | 0.184210526 |
| TCGA-DD-A3A6 | 0.105263158 |
| TCGA-DD-A3A7 | 0.5 |
| TCGA-DD-A3A8 | 0.210526316 |
| TCGA-DD-A3A9 | 0.631578947 |
| TCGA-DD-A4NA | 0.131578947 |
| TCGA-DD-A4NB | 0.236842105 |
| TCGA-DD-A4ND | 0.052631579 |
| TCGA-DD-A4NE | 0.131578947 |
| TCGA-DD-A4NF | 0.236842105 |
| TCGA-DD-A4NG | 0.263157895 |
| TCGA-DD-A4NH | 0.236842105 |
| TCGA-DD-A4NI | 0.394736842 |
| TCGA-DD-A4NJ | 0.263157895 |
| TCGA-DD-A4NK | 0.184210526 |
| TCGA-DD-A4NL | 0.052631579 |
| TCGA-DD-A4NN | 0.184210526 |
| TCGA-DD-A4NO | 0.236842105 |
| TCGA-DD-A4NP | 0.078947368 |
| TCGA-DD-A4NQ | 0.184210526 |
| TCGA-DD-A4NR | 0.263157895 |
| TCGA-DD-A4NS | 0.210526316 |
| TCGA-DD-A4NV | 0.184210526 |
| TCGA-DD-A73A | 0.394736842 |
| TCGA-DD-A73B | 0.157894737 |
| TCGA-DD-A73C | 0.447368421 |
| TCGA-DD-A73D | 0.236842105 |
| TCGA-DD-A73E | 0.342105263 |
| TCGA-DD-A73F | 0.342105263 |
| TCGA-DD-A73G | 0.394736842 |
| TCGA-DD-AA3A | 1.052631579 |
| TCGA-DD-AAC8 | 13.89473684 |
| TCGA-DD-AAC9 | 1.815789474 |
| TCGA-DD-AACA | 2.447368421 |
| TCGA-DD-AACB | 2.473684211 |
| TCGA-DD-AACC | 1.973684211 |
| TCGA-DD-AACD | 2.026315789 |
| TCGA-DD-AACE | 1.842105263 |
| TCGA-DD-AACF | 3.184210526 |
| TCGA-DD-AACG | 2.394736842 |
| TCGA-DD-AACH | 2.052631579 |
| TCGA-DD-AACI | 9.236842105 |
| TCGA-DD-AACJ | 2.105263158 |
| TCGA-DD-AACK | 3.578947368 |
| TCGA-DD-AACL | 8.394736842 |
| TCGA-DD-AACM | 1.289473684 |
| TCGA-DD-AACN | 1.078947368 |
| TCGA-DD-AACO | 0.973684211 |
| TCGA-DD-AACP | 4.263157895 |
| TCGA-DD-AACQ | 5.473684211 |
| TCGA-DD-AACS | 1.131578947 |
| TCGA-DD-AACT | 5.052631579 |
| TCGA-DD-AACU | 2.631578947 |
| TCGA-DD-AACV | 2.184210526 |
| TCGA-DD-AACW | 1.026315789 |
| TCGA-DD-AACX | 3 |
| TCGA-DD-AACY | 2.026315789 |
| TCGA-DD-AACZ | 4.078947368 |
| TCGA-DD-AAD0 | 2.552631579 |
| TCGA-DD-AAD1 | 3.184210526 |
| TCGA-DD-AAD2 | 1.605263158 |
| TCGA-DD-AAD3 | 1.921052632 |
| TCGA-DD-AAD5 | 3.342105263 |
| TCGA-DD-AAD6 | 2.578947368 |
| TCGA-DD-AAD8 | 2.578947368 |
| TCGA-DD-AADA | 2.763157895 |
| TCGA-DD-AADB | 3.184210526 |
| TCGA-DD-AADC | 1.947368421 |
| TCGA-DD-AADD | 2.052631579 |
| TCGA-DD-AADE | 2.894736842 |
| TCGA-DD-AADF | 5.605263158 |
| TCGA-DD-AADG | 4.210526316 |
| TCGA-DD-AADI | 2.657894737 |
| TCGA-DD-AADJ | 1.421052632 |
| TCGA-DD-AADK | 1.421052632 |
| TCGA-DD-AADL | 3.131578947 |
| TCGA-DD-AADM | 4.789473684 |
| TCGA-DD-AADN | 2.736842105 |
| TCGA-DD-AADO | 5.421052632 |
| TCGA-DD-AADP | 2.184210526 |
| TCGA-DD-AADQ | 2.842105263 |
| TCGA-DD-AADR | 2.131578947 |
| TCGA-DD-AADS | 3.605263158 |
| TCGA-DD-AADU | 2.210526316 |
| TCGA-DD-AADV | 2.526315789 |
| TCGA-DD-AADW | 1.289473684 |
| TCGA-DD-AADY | 1.131578947 |
| TCGA-DD-AAE0 | 1.631578947 |
| TCGA-DD-AAE1 | 1.131578947 |
| TCGA-DD-AAE2 | 2.131578947 |
| TCGA-DD-AAE3 | 3.710526316 |
| TCGA-DD-AAE4 | 1.605263158 |
| TCGA-DD-AAE6 | 2.157894737 |
| TCGA-DD-AAE7 | 6.684210526 |
| TCGA-DD-AAE8 | 1.631578947 |
| TCGA-DD-AAE9 | 2.684210526 |
| TCGA-DD-AAEA | 4.368421053 |
| TCGA-DD-AAEB | 3 |
| TCGA-DD-AAED | 1.710526316 |
| TCGA-DD-AAEE | 1.868421053 |
| TCGA-DD-AAEG | 2.552631579 |
| TCGA-DD-AAEH | 2.342105263 |
| TCGA-DD-AAEI | 2.868421053 |
| TCGA-DD-AAEK | 1.894736842 |
| TCGA-DD-AAVP | 1.973684211 |
| TCGA-DD-AAVQ | 1.342105263 |
| TCGA-DD-AAVR | 1.605263158 |
| TCGA-DD-AAVS | 1.131578947 |
| TCGA-DD-AAVU | 1.447368421 |
| TCGA-DD-AAVV | 3.131578947 |
| TCGA-DD-AAVW | 0.657894737 |
| TCGA-DD-AAVX | 1.736842105 |
| TCGA-DD-AAVY | 2.263157895 |
| TCGA-DD-AAVZ | 1.105263158 |
| TCGA-DD-AAW0 | 2.184210526 |
| TCGA-DD-AAW1 | 2.605263158 |
| TCGA-DD-AAW2 | 2.815789474 |
| TCGA-DD-AAW3 | 2.342105263 |
| TCGA-ED-A459 | 0.894736842 |
| TCGA-ED-A4XI | 0.605263158 |
| TCGA-ED-A5KG | 0.052631579 |
| TCGA-ED-A66X | 0.105263158 |
| TCGA-ED-A66Y | 0.263157895 |
| TCGA-ED-A7PX | 0.184210526 |
| TCGA-ED-A7PY | 0.131578947 |
| TCGA-ED-A7PZ | 0.447368421 |
| TCGA-ED-A7XO | 0.157894737 |
| TCGA-ED-A7XP | 0.236842105 |
| TCGA-ED-A82E | 0.236842105 |
| TCGA-ED-A8O5 | 2.789473684 |
| TCGA-ED-A8O6 | 1.552631579 |
| TCGA-ED-A97K | 0.894736842 |
| TCGA-EP-A12J | 0.289473684 |
| TCGA-EP-A26S | 0.210526316 |
| TCGA-EP-A2KA | 0.289473684 |
| TCGA-EP-A2KB | 0.131578947 |
| TCGA-EP-A2KC | 0.315789474 |
| TCGA-EP-A3JL | 0.210526316 |
| TCGA-EP-A3RK | 0.105263158 |
| TCGA-ES-A2HS | 0.157894737 |
| TCGA-ES-A2HT | 0.210526316 |
| TCGA-FV-A23B | 0.473684211 |
| TCGA-FV-A2QQ | 0.631578947 |
| TCGA-FV-A2QR | 0.263157895 |
| TCGA-FV-A3I0 | 0.236842105 |
| TCGA-FV-A3I1 | 0.184210526 |
| TCGA-FV-A3R2 | 0.131578947 |
| TCGA-FV-A3R3 | 0.184210526 |
| TCGA-FV-A495 | 0.263157895 |
| TCGA-FV-A496 | 0.447368421 |
| TCGA-FV-A4ZP | 0.315789474 |
| TCGA-FV-A4ZQ | 0.131578947 |
| TCGA-G3-A25S | 0.552631579 |
| TCGA-G3-A25T | 0.210526316 |
| TCGA-G3-A25U | 0.210526316 |
| TCGA-G3-A25V | 0.263157895 |
| TCGA-G3-A25W | 0.447368421 |
| TCGA-G3-A25Y | 0.315789474 |
| TCGA-G3-A25Z | 0.315789474 |
| TCGA-G3-A3CG | 2.5 |
| TCGA-G3-A3CH | 0.263157895 |
| TCGA-G3-A3CI | 0.052631579 |
| TCGA-G3-A3CK | 0.578947368 |
| TCGA-G3-A5SI | 0.157894737 |
| TCGA-G3-A5SJ | 0.184210526 |
| TCGA-G3-A5SK | 0.289473684 |
| TCGA-G3-A5SL | 0.368421053 |
| TCGA-G3-A5SM | 0.157894737 |
| TCGA-G3-A6UC | 0.447368421 |
| TCGA-G3-A7M5 | 0.868421053 |
| TCGA-G3-A7M6 | 0.368421053 |
| TCGA-G3-A7M7 | 0.210526316 |
| TCGA-G3-A7M8 | 0.315789474 |
| TCGA-G3-A7M9 | 0.473684211 |
| TCGA-G3-AAUZ | 1.868421053 |
| TCGA-G3-AAV0 | 4.078947368 |
| TCGA-G3-AAV1 | 1.289473684 |
| TCGA-G3-AAV2 | 1.026315789 |
| TCGA-G3-AAV3 | 2.184210526 |
| TCGA-G3-AAV4 | 2.342105263 |
| TCGA-G3-AAV5 | 1.763157895 |
| TCGA-G3-AAV6 | 1.789473684 |
| TCGA-G3-AAV7 | 2 |
| TCGA-GJ-A3OU | 1.342105263 |
| TCGA-GJ-A6C0 | 0.210526316 |
| TCGA-GJ-A9DB | 2 |
| TCGA-HP-A5MZ | 0.157894737 |
| TCGA-HP-A5N0 | 0.578947368 |
| TCGA-K7-A5RF | 0.184210526 |
| TCGA-K7-A5RG | 0.184210526 |
| TCGA-K7-A6G5 | 0.263157895 |
| TCGA-K7-AAU7 | 1.578947368 |
| TCGA-KR-A7K0 | 0.289473684 |
| TCGA-KR-A7K2 | 0.105263158 |
| TCGA-KR-A7K7 | 0.210526316 |
| TCGA-KR-A7K8 | 0.131578947 |
| TCGA-LG-A6GG | 0.315789474 |
| TCGA-LG-A9QC | 0.973684211 |
| TCGA-LG-A9QD | 2.605263158 |
| TCGA-MI-A75C | 0.342105263 |
| TCGA-MI-A75E | 0.552631579 |
| TCGA-MI-A75G | 1.052631579 |
| TCGA-MI-A75H | 0.526315789 |
| TCGA-MI-A75I | 0.684210526 |
| TCGA-MR-A520 | 0.5 |
| TCGA-MR-A8JO | 0.789473684 |
| TCGA-NI-A4U2 | 0.263157895 |
| TCGA-NI-A8LF | 2.526315789 |
| TCGA-O8-A75V | 0.210526316 |
| TCGA-PD-A5DF | 0.236842105 |
| TCGA-QA-A7B7 | 0.157894737 |
| TCGA-RC-A6M3 | 1.921052632 |
| TCGA-RC-A6M4 | 4.289473684 |
| TCGA-RC-A6M5 | 0.578947368 |
| TCGA-RC-A6M6 | 4.473684211 |
| TCGA-RC-A7S9 | 0.210526316 |
| TCGA-RC-A7SB | 1.526315789 |
| TCGA-RC-A7SF | 0.236842105 |
| TCGA-RC-A7SH | 1.578947368 |
| TCGA-RC-A7SK | 0.605263158 |
| TCGA-RG-A7D4 | 0.526315789 |
| TCGA-T1-A6J8 | 2.026315789 |
| TCGA-UB-A7MA | 0.315789474 |
| TCGA-UB-A7MB | 2.078947368 |
| TCGA-UB-A7MC | 0.315789474 |
| TCGA-UB-A7MD | 0.236842105 |
| TCGA-UB-A7ME | 0.157894737 |
| TCGA-UB-A7MF | 0.263157895 |
| TCGA-UB-AA0U | 1.789473684 |
| TCGA-UB-AA0V | 1.026315789 |
| TCGA-WJ-A86L | 3.105263158 |
| TCGA-WQ-A9G7 | 9.184210526 |
| TCGA-WQ-AB4B | 1.473684211 |
| TCGA-WX-AA44 | 1.868421053 |
| TCGA-WX-AA46 | 1.473684211 |
| TCGA-WX-AA47 | 0.842105263 |
| TCGA-XR-A8TC | 1.157894737 |
| TCGA-XR-A8TD | 1.5 |
| TCGA-XR-A8TE | 0.263157895 |
| TCGA-XR-A8TF | 3.5 |
| TCGA-XR-A8TG | 2.157894737 |
| TCGA-YA-A8S7 | 1.631578947 |
| TCGA-ZP-A9CV | 2.763157895 |
| TCGA-ZP-A9CY | 2.052631579 |
| TCGA-ZP-A9CZ | 1.078947368 |
| TCGA-ZP-A9D0 | 1.315789474 |
| TCGA-ZP-A9D1 | 3.5 |
| TCGA-ZP-A9D2 | 1.184210526 |
| TCGA-ZP-A9D4 | 1.447368421 |
| TCGA-ZS-A9CD | 2.342105263 |
| TCGA-ZS-A9CE | 3.026315789 |
| TCGA-ZS-A9CF | 2.289473684 |
| TCGA-ZS-A9CG | 1.868421053 |

*HCC: hepatocellular carcinoma, TMB, tumor mutation burden
