# Supplementary material for: Prognostic analysis of tumor mutation burden and immune infiltration in hepatocellular carcinoma based on TCGA data
Source: Aging (Albany NY). 2021 Apr 4;13(8):11257–80. doi: 10.18632/aging.202811 (PMC8109113; doi:10.18632/aging.202811)
Supplement: Supplementary Table 2 [file aging-13-202811-s002.doc]

**Supplementary Table 2. Differentially expressed genes analysis between two TMB groups.**

| **gene** | **logFC** | **AveExpr** | **t** | **PValue** | **FDR** | **B** |
| --- | --- | --- | --- | --- | --- | --- |
| EDIL3 | -0.878848397 | 1.847534968 | -5.34544594 | 1.63E-07 | 0.001033796 | 6.819994586 |
| CYP2D6 | 1.177975757 | 6.867180319 | 4.971783915 | 1.04E-06 | 0.001377501 | 5.256501039 |
| NOX4 | -0.587287795 | 0.133544367 | -4.963079372 | 1.08E-06 | 0.001377501 | 4.746340186 |
| CYP2D7 | 1.075180687 | 3.088447542 | 4.854828457 | 1.82E-06 | 0.001775934 | 4.762249922 |
| AKT3 | -0.607434369 | 2.631513743 | -4.752570328 | 2.93E-06 | 0.002393827 | 4.310728354 |
| PRKG1 | -0.65328102 | 0.757431678 | -4.770453439 | 2.70E-06 | 0.002393827 | 4.130797276 |
| DDTL | 0.640123055 | 2.164472103 | 4.693828909 | 3.85E-06 | 0.00287812 | 4.038065165 |
| HCN3 | 0.618396564 | 5.016905659 | 4.544949802 | 7.57E-06 | 0.00481098 | 3.383251256 |
| SEC16B | 0.597185612 | 1.443806835 | 4.526695816 | 8.21E-06 | 0.004971958 | 3.291790933 |
| SPP2 | 1.529450918 | 4.70279756 | 4.477700732 | 1.02E-05 | 0.005410063 | 3.118833565 |
| CECR2 | 0.885801206 | 2.909637052 | 4.435509392 | 1.23E-05 | 0.005792958 | 3.01246201 |
| ENDOD1 | -0.60478279 | 3.018893649 | -4.25223907 | 2.72E-05 | 0.009626469 | 2.290890733 |
| PCDH18 | -0.626990646 | 0.539172969 | -4.243239692 | 2.82E-05 | 0.009626469 | 2.098448092 |
| C17orf51 | -0.643851615 | 0.850794722 | -4.222796077 | 3.08E-05 | 0.009847801 | 2.069937688 |
| SEMA6D | -0.645562985 | 0.522960564 | -4.12943998 | 4.55E-05 | 0.011799586 | 1.695434812 |
| DDC | 0.956347474 | 4.682196002 | 4.10776628 | 4.97E-05 | 0.012084513 | 1.646248955 |
| MLLT3 | -0.716600034 | 1.070612979 | -4.070210161 | 5.80E-05 | 0.01303106 | 1.553213459 |
| TNC | -0.771166986 | 3.363794582 | -4.043438854 | 6.48E-05 | 0.013736371 | 1.485942686 |
| OLFML1 | -0.672625475 | 2.408511408 | -4.026037583 | 6.95E-05 | 0.013736371 | 1.456847787 |
| TLX1 | 0.99464823 | 0.741186792 | 4.033858485 | 6.73E-05 | 0.013736371 | 1.39539991 |
| KIT | -0.621921663 | 0.329770378 | -4.044710314 | 6.44E-05 | 0.013736371 | 1.378101792 |
| CYP3A43 | 1.074795487 | 0.356162847 | 4.026820534 | 6.93E-05 | 0.013736371 | 1.323137424 |
| CLIP2 | -0.632462135 | 3.664391746 | -4.015831539 | 7.25E-05 | 0.013749958 | 1.36276076 |
| NAT8 | 0.847916652 | 4.741889395 | 4.011730841 | 7.37E-05 | 0.013774747 | 1.280167562 |
| SLC25A24 | -0.70169607 | 1.802675588 | -4.004053976 | 7.60E-05 | 0.013806611 | 1.369056989 |
| COL12A1 | -0.653195229 | 3.087529882 | -3.982767925 | 8.28E-05 | 0.013806611 | 1.277876588 |
| LTBP1 | -0.628119888 | 3.85096814 | -3.979701476 | 8.38E-05 | 0.013806611 | 1.215759603 |
| GALNT7 | -0.669764897 | 0.902592623 | -3.977165147 | 8.47E-05 | 0.013806611 | 1.215207319 |
| ADAMTS12 | -0.598316634 | 1.388540769 | -3.97100341 | 8.68E-05 | 0.013870809 | 1.233118545 |
| COL3A1 | -0.789352062 | 7.131839093 | -3.885949968 | 1.22E-04 | 0.016477264 | 0.844404719 |
| TANC2 | -0.631112304 | 1.694499266 | -3.838211715 | 1.47E-04 | 0.018681971 | 0.790610269 |
| DUSP4 | -0.669823743 | 1.425848625 | -3.803290436 | 1.68E-04 | 0.019469593 | 0.664035837 |
| CYP3A5 | 0.692531044 | 7.341082644 | 3.78918489 | 1.78E-04 | 0.019827575 | 0.505825439 |
| LIMCH1 | -0.618785352 | 2.991213254 | -3.769474249 | 1.92E-04 | 0.020495198 | 0.52651518 |
| MMRN1 | -0.607480202 | 0.75568357 | -3.750432825 | 2.06E-04 | 0.021161543 | 0.450893124 |
| PDE1A | -0.671387291 | 0.588034803 | -3.728227921 | 2.25E-04 | 0.022390286 | 0.366332317 |
| NPIPB15 | 0.931690024 | 0.832219725 | 3.713464671 | 2.38E-04 | 0.022832238 | 0.339422011 |
| ANTXR1 | -0.650095673 | 3.245616128 | -3.712177288 | 2.39E-04 | 0.022832238 | 0.311284456 |
| POSTN | -1.069896714 | 2.477265077 | -3.697587229 | 2.52E-04 | 0.022951552 | 0.311325667 |
| KCNE4 | -0.676224914 | 1.174420636 | -3.68039569 | 2.69E-04 | 0.023786518 | 0.250912257 |
| IGSF23 | 0.914584156 | 2.90236781 | 3.661352268 | 2.89E-04 | 0.024371074 | 0.162045838 |
| PFKP | -0.692632414 | 3.032512395 | -3.629517893 | 3.26E-04 | 0.025281328 | 0.048577884 |
| CAPN12 | 0.672434488 | 3.144042386 | 3.630080665 | 3.25E-04 | 0.025281328 | 0.036578079 |
| TTR | 0.938405557 | 10.02747642 | 3.616335905 | 3.43E-04 | 0.025521956 | 0.028548393 |
| SERPINF2 | 0.660433058 | 9.681053064 | 3.618939348 | 3.39E-04 | 0.025521956 | 0.025054301 |
| FTCD | 0.881887147 | 7.481961779 | 3.618655107 | 3.40E-04 | 0.025521956 | -0.07642235 |
| APOA2 | 0.909688065 | 12.69991011 | 3.601623709 | 3.62E-04 | 0.025739798 | 0.003595551 |
| TDGF1 | 1.098711908 | 1.221295024 | 3.601166978 | 3.62E-04 | 0.025739798 | 0.001359029 |
| SCD5 | -0.724384169 | 0.619396988 | -3.601229512 | 3.62E-04 | 0.025739798 | -0.02911831 |
| APOM | 0.720207246 | 7.610076381 | 3.584151478 | 3.86E-04 | 0.026477404 | -0.186875029 |
| COL8A1 | -0.690837276 | 1.957534969 | -3.560692302 | 4.21E-04 | 0.026732276 | -0.123220052 |
| TMEM45A | -0.867075454 | 3.754786791 | -3.571393091 | 4.05E-04 | 0.026732276 | -0.203134191 |
| ABCG5 | 0.749592144 | 4.938890465 | 3.565442112 | 4.13E-04 | 0.026732276 | -0.309868031 |
| DGAT2 | 0.601637704 | 6.811043493 | 3.551430294 | 4.35E-04 | 0.026869331 | -0.334024631 |
| PMEPA1 | -0.70128406 | 3.085187371 | -3.525230312 | 4.79E-04 | 0.028869859 | -0.299311363 |
| LUM | -1.213781508 | 3.209643694 | -3.516618773 | 4.94E-04 | 0.028978044 | -0.32895626 |
| SLC7A9 | 0.648032265 | 3.712246057 | 3.493951422 | 5.37E-04 | 0.029671454 | -0.466076079 |
| NR1I3 | 0.827595222 | 5.006234308 | 3.49786509 | 5.29E-04 | 0.029671454 | -0.536067194 |
| EXOC3L4 | 0.657369929 | 4.115098006 | 3.482133329 | 5.60E-04 | 0.030178907 | -0.539686479 |
| CDH11 | -0.720702954 | 1.806257581 | -3.47697894 | 5.71E-04 | 0.030486848 | -0.38435859 |
| SSC5D | -0.678644777 | 0.43136727 | -3.472114973 | 5.81E-04 | 0.030639563 | -0.430919962 |
| SLIT2 | -0.725487756 | 0.744964541 | -3.466093116 | 5.93E-04 | 0.030799473 | -0.432689897 |
| CCL15 | 0.633763416 | 3.846213 | 3.462395123 | 6.01E-04 | 0.031014845 | -0.581102148 |
| COL1A2 | -0.654798745 | 6.995466251 | -3.448906821 | 6.31E-04 | 0.03147397 | -0.661361453 |
| LOX | -0.585847404 | 2.346673388 | -3.44630342 | 6.37E-04 | 0.03164458 | -0.499764577 |
| GNMT | 0.960675065 | 4.645856186 | 3.437468862 | 6.58E-04 | 0.031790723 | -0.717483599 |
| LGALS3 | -0.588336312 | 5.112815937 | -3.438344033 | 6.56E-04 | 0.031790723 | -0.730263868 |
| SULF1 | -0.816508937 | 2.753729469 | -3.420424914 | 6.99E-04 | 0.032287204 | -0.606182435 |
| GPAM | 0.609186791 | 7.081383411 | 3.417351035 | 7.06E-04 | 0.032310252 | -0.760580043 |
| AKR1D1 | 1.080514297 | 4.265879811 | 3.414784679 | 7.13E-04 | 0.032419278 | -0.764658334 |
| APCDD1 | -0.942165688 | 0.903299151 | -3.406077139 | 7.35E-04 | 0.03268878 | -0.605915267 |
| CRYBG3 | -0.689595471 | 1.116132386 | -3.391490653 | 7.74E-04 | 0.033256518 | -0.644038454 |
| LPA | 0.876370578 | 3.55178398 | 3.387130344 | 7.86E-04 | 0.033256518 | -0.792224253 |
| PRODH2 | 0.852730595 | 5.567132218 | 3.386754799 | 7.87E-04 | 0.033256518 | -0.903996678 |
| EPHA3 | -0.742369738 | 1.058518007 | -3.350381289 | 8.95E-04 | 0.035494698 | -0.766239143 |
| SLC2A2 | 0.751152615 | 7.884449627 | 3.342857172 | 9.18E-04 | 0.035494698 | -0.954469424 |
| ARSE | 0.872438277 | 5.994376809 | 3.326738358 | 9.71E-04 | 0.03676321 | -1.088820719 |
| ACSL6 | 0.797998739 | 2.405150742 | 3.318967105 | 9.98E-04 | 0.037265273 | -0.897835288 |
| VCAN | -0.856978531 | 3.417631757 | -3.315221349 | 0.00101113 | 0.037265273 | -0.993355452 |
| RHBG | 1.343041682 | 2.094254442 | 3.309629001 | 0.001030918 | 0.037578006 | -0.905553802 |
| COL1A1 | -0.729517305 | 7.552296681 | -3.30541455 | 0.001046068 | 0.03800215 | -1.088681333 |
| PCP4L1 | 0.827304487 | 0.276906961 | 3.296039243 | 0.001080514 | 0.038376362 | -0.945344334 |
| THBS2 | -0.846701632 | 3.938374473 | -3.279125722 | 0.001145336 | 0.039359302 | -1.156256754 |
| NID2 | -0.586794165 | 1.76655283 | -3.277300193 | 0.001152544 | 0.039394068 | -0.987659253 |
| SERPINA4 | 0.802152696 | 7.148669208 | 3.272239681 | 0.001172746 | 0.039707143 | -1.213515977 |
| DCN | -0.960990816 | 4.275931682 | -3.267891083 | 0.001190368 | 0.040041091 | -1.217719329 |
| SLC38A3 | 0.622289473 | 8.671045713 | 3.255952897 | 0.001240018 | 0.040726122 | -1.178509445 |
| ARMCX3 | -0.629432991 | 3.692129733 | -3.251600391 | 0.001258592 | 0.041033322 | -1.221449533 |
| SPDYC | 0.674209655 | 0.132235096 | 3.249836493 | 0.001266192 | 0.041070504 | -1.080491472 |
| MOGAT3 | 0.788760583 | 3.722164562 | 3.240037546 | 0.001309197 | 0.041825214 | -1.266485086 |
| GRAMD1B | -0.775672763 | 1.749858323 | -3.233527362 | 0.001338512 | 0.04247306 | -1.114635114 |
| HOXA13 | 1.177948777 | 0.609264361 | 3.232334544 | 0.001343948 | 0.042487247 | -1.11213174 |
| MOXD1 | -0.82824136 | 0.602484821 | -3.23166964 | 0.001346988 | 0.042487247 | -1.114824881 |
| ENPP7 | 0.850408085 | 3.399237967 | 3.227424766 | 0.001366543 | 0.042587229 | -1.269899286 |
| APOA1 | 0.885702512 | 11.75683425 | 3.208353336 | 0.001457686 | 0.044265323 | -1.189109765 |
| TUSC3 | -0.714909453 | 2.089911929 | -3.211556683 | 0.001441994 | 0.044265323 | -1.19435759 |
| FABP1 | 0.992341895 | 8.867867864 | 3.195064008 | 0.001524488 | 0.044870066 | -1.349094333 |
| SERPINC1 | 0.861754343 | 10.60564538 | 3.189274914 | 0.001554465 | 0.045347594 | -1.279616333 |
| DHRS2 | 1.217290553 | 3.357587237 | 3.18849497 | 0.001558545 | 0.045347594 | -1.375792785 |
| EDA | -0.661811946 | 1.289549424 | -3.156062083 | 0.00173724 | 0.047339073 | -1.323117572 |
| PANX2 | 0.722489712 | 3.526322969 | 3.155171184 | 0.001742406 | 0.047339073 | -1.500591028 |
| CES3 | 0.64606894 | 4.356361046 | 3.1537238 | 0.001750828 | 0.047466474 | -1.584388373 |
| SLC22A25 | 0.739258589 | 2.817433507 | 3.149861067 | 0.00177349 | 0.047543281 | -1.437272667 |
| TC2N | -0.764330327 | 2.138491284 | -3.140045493 | 0.001832299 | 0.047740464 | -1.403506168 |
| CDHR5 | 0.765139419 | 7.185361787 | 3.140544415 | 0.001829267 | 0.047740464 | -1.61014835 |
| SHBG | 0.804506593 | 3.369516753 | 3.13811638 | 0.001844067 | 0.047741397 | -1.532508585 |
| ABCG8 | 0.798489344 | 5.193953411 | 3.129343394 | 0.001898467 | 0.048374763 | -1.695479504 |
| PTN | -0.611058352 | 0.211019861 | -3.128559775 | 0.001903397 | 0.048403395 | -1.406258731 |
| A1BG | 0.749516212 | 5.740665261 | 3.112559621 | 0.002006687 | 0.04980113 | -1.747250913 |

*Gene: gene name; logFC: log2 logarithm of the difference multiple between groups;AveExpr: Average gene expression;T: T-test value;PValue: significance test P value, FDR: P value for multiple hypothesis verification;B: Bayesian statistics
