# Supplementary material for: Prognostic analysis of tumor mutation burden and immune infiltration in hepatocellular carcinoma based on TCGA data
Source: Aging (Albany NY). 2021 Apr 4;13(8):11257–80. doi: 10.18632/aging.202811 (PMC8109113; doi:10.18632/aging.202811)
Supplement: Supplementary Table 3 [file aging-13-202811-s003.doc]

**Supplementary Table 3. Top GO items for differentially expressed genes.**

| **ID** | **Description** | **ont_type** | **GeneRatio** | **BgRatio** | **pvalue** | **p.adjust** | **qvalue** | **geneID** |
| --- | --- | --- | --- | --- | --- | --- | --- | --- |
| GO:0005201 | extracellular matrix structural constituent | MF | 15/97 | 155/17632 | 6.06E-15 | 1.82E-12 | 1.49E-12 | EDIL3/TNC/COL12A1/LTBP1/COL3A1/MMRN1/POSTN/COL8A1/LUM/COL1A2/VCAN/COL1A1/THBS2/NID2/DCN |
| GO:0005539 | glycosaminoglycan binding | MF | 10/97 | 222/17632 | 3.96E-07 | 5.95E-05 | 4.87E-05 | POSTN/SLIT2/CCL15/SULF1/LPA/VCAN/THBS2/DCN/SERPINC1/PTN |
| GO:0030020 | extracellular matrix structural constituent conferring tensile strength | MF | 5/97 | 37/17632 | 1.72E-06 | 0.000172759 | 0.000141373 | COL12A1/COL3A1/COL8A1/COL1A2/COL1A1 |
| GO:0017127 | cholesterol transporter activity | MF | 4/97 | 19/17632 | 3.13E-06 | 0.000235613 | 0.000192808 | APOA2/ABCG5/APOA1/ABCG8 |
| GO:0070330 | aromatase activity | MF | 4/97 | 22/17632 | 5.83E-06 | 0.000351253 | 0.000287439 | CYP2D6/CYP2D7/CYP3A43/CYP3A5 |
| GO:0004497 | monooxygenase activity | MF | 6/97 | 98/17632 | 1.66E-05 | 0.000832871 | 0.000681559 | CYP2D6/CYP2D7/CYP3A43/CYP3A5/AKR1D1/MOXD1 |
| GO:0048407 | platelet-derived growth factor binding | MF | 3/97 | 11/17632 | 2.58E-05 | 0.000887454 | 0.000726226 | COL3A1/COL1A2/COL1A1 |
| GO:0016712 | oxidoreductase activity, acting on paired donors, with incorporation or reduction of molecular oxygen, reduced flavin or flavoprotein as one donor, and incorporation of one atom of oxygen | MF | 4/97 | 32/17632 | 2.75E-05 | 0.000887454 | 0.000726226 | CYP2D6/CYP2D7/CYP3A43/CYP3A5 |
| GO:0016705 | oxidoreductase activity, acting on paired donors, with incorporation or reduction of molecular oxygen | MF | 7/97 | 161/17632 | 3.00E-05 | 0.000887454 | 0.000726226 | CYP2D6/CYP2D7/CYP3A43/CYP3A5/SCD5/AKR1D1/MOXD1 |
| GO:0015248 | sterol transporter activity | MF | 4/97 | 33/17632 | 3.12E-05 | 0.000887454 | 0.000726226 | APOA2/ABCG5/APOA1/ABCG8 |
| GO:0008201 | heparin binding | MF | 7/97 | 163/17632 | 3.24E-05 | 0.000887454 | 0.000726226 | POSTN/SLIT2/CCL15/LPA/THBS2/SERPINC1/PTN |
| GO:0004857 | enzyme inhibitor activity | MF | 10/97 | 371/17632 | 3.73E-05 | 0.000935515 | 0.000765555 | SPP2/SERPINF2/APOA2/SLIT2/LGALS3/LPA/SERPINA4/APOA1/SERPINC1/PTN |
| GO:1901681 | sulfur compound binding | MF | 8/97 | 242/17632 | 5.67E-05 | 0.001311838 | 0.00107351 | POSTN/SLIT2/CCL15/GNMT/LPA/THBS2/SERPINC1/PTN |
| GO:0002020 | protease binding | MF | 6/97 | 125/17632 | 6.57E-05 | 0.001411948 | 0.001155432 | KIT/COL3A1/SERPINF2/COL1A2/COL1A1/SERPINC1 |
| GO:0005319 | lipid transporter activity | MF | 6/97 | 146/17632 | 0.000154722 | 0.003104764 | 0.002540706 | APOA2/APOM/ABCG5/APOA1/FABP1/ABCG8 |
| GO:0030021 | extracellular matrix structural constituent conferring compression resistance | MF | 3/97 | 22/17632 | 0.000230387 | 0.004334158 | 0.003546749 | LUM/VCAN/DCN |
| GO:0050840 | extracellular matrix binding | MF | 4/97 | 56/17632 | 0.000253894 | 0.004495424 | 0.003678717 | SSC5D/SLIT2/LGALS3/DCN |
| GO:0005518 | collagen binding | MF | 4/97 | 67/17632 | 0.000505936 | 0.008421658 | 0.006891653 | ANTXR1/LUM/NID2/DCN |
| GO:0043236 | laminin binding | MF | 3/97 | 29/17632 | 0.0005316 | 0.008421658 | 0.006891653 | SSC5D/SLIT2/LGALS3 |
| GO:0043394 | proteoglycan binding | MF | 3/97 | 32/17632 | 0.000713032 | 0.010731131 | 0.008781552 | TNC/SLIT2/PTN |
| GO:0043062 | extracellular structure organization | BP | 20/98 | 402/18493 | 2.51E-14 | 4.71E-11 | 3.88E-11 | TNC/COL12A1/COL3A1/ANTXR1/POSTN/TTR/SERPINF2/APOA2/APOM/COL8A1/LUM/COL1A2/LOX/SULF1/LPA/VCAN/COL1A1/NID2/DCN/APOA1 |
| GO:0030198 | extracellular matrix organization | BP | 16/98 | 348/18493 | 4.11E-11 | 3.84E-08 | 3.17E-08 | TNC/COL12A1/COL3A1/ANTXR1/POSTN/TTR/SERPINF2/COL8A1/LUM/COL1A2/LOX/SULF1/VCAN/COL1A1/NID2/DCN |
| GO:0031589 | cell-substrate adhesion | BP | 14/98 | 338/18493 | 2.90E-09 | 1.81E-06 | 1.49E-06 | EDIL3/ADAMTS12/COL3A1/LIMCH1/MMRN1/ANTXR1/POSTN/COL8A1/EPHA3/COL1A1/NID2/APOA1/EDA/PTN |
| GO:0030199 | collagen fibril organization | BP | 7/98 | 52/18493 | 1.04E-08 | 4.86E-06 | 4.01E-06 | COL12A1/COL3A1/SERPINF2/LUM/COL1A2/LOX/COL1A1 |
| GO:0050892 | intestinal absorption | BP | 6/98 | 35/18493 | 2.72E-08 | 1.02E-05 | 8.39E-06 | APOA2/ABCG5/SLC2A2/APOA1/FABP1/ABCG8 |
| GO:0030299 | intestinal cholesterol absorption | BP | 4/98 | 15/18493 | 9.68E-07 | 0.000301788 | 0.000248738 | APOA2/ABCG5/APOA1/ABCG8 |
| GO:0098856 | intestinal lipid absorption | BP | 4/98 | 16/18493 | 1.29E-06 | 0.000343499 | 0.000283117 | APOA2/ABCG5/APOA1/ABCG8 |
| GO:0010810 | regulation of cell-substrate adhesion | BP | 9/98 | 209/18493 | 1.67E-06 | 0.000347952 | 0.000286787 | EDIL3/LIMCH1/MMRN1/POSTN/COL8A1/EPHA3/COL1A1/APOA1/PTN |
| GO:0044241 | lipid digestion | BP | 4/98 | 17/18493 | 1.67E-06 | 0.000347952 | 0.000286787 | APOA2/ABCG5/APOA1/ABCG8 |
| GO:0002576 | platelet degranulation | BP | 7/98 | 128/18493 | 5.29E-06 | 0.000990689 | 0.00081654 | SPP2/ENDOD1/MMRN1/SERPINF2/SERPINA4/APOA1/A1BG |
| GO:0007586 | digestion | BP | 7/98 | 135/18493 | 7.52E-06 | 0.001279823 | 0.001054848 | APOA2/ABCG5/AKR1D1/SLC2A2/APOA1/FABP1/ABCG8 |
| GO:0042632 | cholesterol homeostasis | BP | 6/98 | 92/18493 | 9.37E-06 | 0.001431236 | 0.001179645 | APOA2/APOM/ABCG5/DGAT2/APOA1/ABCG8 |
| GO:0055092 | sterol homeostasis | BP | 6/98 | 93/18493 | 9.97E-06 | 0.001431236 | 0.001179645 | APOA2/APOM/ABCG5/DGAT2/APOA1/ABCG8 |
| GO:0055088 | lipid homeostasis | BP | 7/98 | 144/18493 | 1.15E-05 | 0.001431236 | 0.001179645 | APOA2/APOM/ABCG5/DGAT2/GPAM/APOA1/ABCG8 |
| GO:0033344 | cholesterol efflux | BP | 5/98 | 57/18493 | 1.27E-05 | 0.001431236 | 0.001179645 | APOA2/APOM/ABCG5/APOA1/ABCG8 |
| GO:0022600 | digestive system process | BP | 6/98 | 97/18493 | 1.27E-05 | 0.001431236 | 0.001179645 | APOA2/ABCG5/SLC2A2/APOA1/FABP1/ABCG8 |
| GO:0034368 | protein-lipid complex remodeling | BP | 4/98 | 28/18493 | 1.38E-05 | 0.001431236 | 0.001179645 | APOA2/APOM/LPA/APOA1 |
| GO:0034369 | plasma lipoprotein particle remodeling | BP | 4/98 | 28/18493 | 1.38E-05 | 0.001431236 | 0.001179645 | APOA2/APOM/LPA/APOA1 |
| GO:0071295 | cellular response to vitamin | BP | 4/98 | 29/18493 | 1.59E-05 | 0.001499188 | 0.001235652 | TNC/POSTN/COL1A1/PTN |
| GO:0006641 | triglyceride metabolic process | BP | 6/98 | 101/18493 | 1.60E-05 | 0.001499188 | 0.001235652 | APOA2/DGAT2/GPAM/MOGAT3/APOA1/FABP1 |
| GO:0062023 | collagen-containing extracellular matrix | CC | 23/107 | 332/19659 | 3.18E-19 | 5.72E-17 | 4.78E-17 | EDIL3/SPP2/TNC/COL12A1/LTBP1/ADAMTS12/COL3A1/MMRN1/POSTN/SERPINF2/COL8A1/LUM/COL1A2/LGALS3/VCAN/COL1A1/THBS2/NID2/DCN/APOA1/SERPINC1/PTN/A1BG |
| GO:0031012 | extracellular matrix | CC | 25/107 | 490/19659 | 1.13E-17 | 1.02E-15 | 8.49E-16 | EDIL3/SPP2/TNC/COL12A1/LTBP1/ADAMTS12/COL3A1/MMRN1/POSTN/SERPINF2/COL8A1/LUM/SSC5D/COL1A2/LOX/LGALS3/VCAN/COL1A1/THBS2/NID2/DCN/APOA1/SERPINC1/PTN/A1BG |
| GO:0044420 | extracellular matrix component | CC | 8/107 | 49/19659 | 2.21E-10 | 1.33E-08 | 1.11E-08 | TNC/COL12A1/LTBP1/COL3A1/COL8A1/LUM/COL1A2/COL1A1 |
| GO:0005788 | endoplasmic reticulum lumen | CC | 14/107 | 301/19659 | 9.77E-10 | 3.86E-08 | 3.23E-08 | SPP2/TNC/COL12A1/LTBP1/COL3A1/APOA2/COL8A1/COL1A2/ARSE/VCAN/COL1A1/APOA1/SERPINC1/CES3 |
| GO:0005581 | collagen trimer | CC | 9/107 | 87/19659 | 1.07E-09 | 3.86E-08 | 3.23E-08 | COL12A1/COL3A1/COL8A1/LUM/COL1A2/LOX/COL1A1/DCN/EDA |
| GO:0098644 | complex of collagen trimers | CC | 5/107 | 19/19659 | 4.76E-08 | 1.43E-06 | 1.19E-06 | COL3A1/COL8A1/LUM/COL1A2/COL1A1 |
| GO:0005583 | fibrillar collagen trimer | CC | 4/107 | 11/19659 | 2.66E-07 | 5.98E-06 | 5.00E-06 | COL3A1/LUM/COL1A2/COL1A1 |
| GO:0098643 | banded collagen fibril | CC | 4/107 | 11/19659 | 2.66E-07 | 5.98E-06 | 5.00E-06 | COL3A1/LUM/COL1A2/COL1A1 |
| GO:0045177 | apical part of cell | CC | 10/107 | 373/19659 | 3.68E-05 | 0.000735615 | 0.000615163 | NOX4/KCNE4/TDGF1/ABCG5/SLC7A9/SLC2A2/FABP1/EDA/CDHR5/ABCG8 |
| GO:0034358 | plasma lipoprotein particle | CC | 4/107 | 38/19659 | 5.31E-05 | 0.000868817 | 0.000726554 | APOA2/APOM/LPA/APOA1 |
| GO:1990777 | lipoprotein particle | CC | 4/107 | 38/19659 | 5.31E-05 | 0.000868817 | 0.000726554 | APOA2/APOM/LPA/APOA1 |
| GO:0032994 | protein-lipid complex | CC | 4/107 | 40/19659 | 6.52E-05 | 0.000977827 | 0.000817715 | APOA2/APOM/LPA/APOA1 |
| GO:0034361 | very-low-density lipoprotein particle | CC | 3/107 | 20/19659 | 0.000167056 | 0.002147868 | 0.00179617 | APOA2/APOM/APOA1 |
| GO:0034385 | triglyceride-rich plasma lipoprotein particle | CC | 3/107 | 20/19659 | 0.000167056 | 0.002147868 | 0.00179617 | APOA2/APOM/APOA1 |
| GO:0016324 | apical plasma membrane | CC | 8/107 | 308/19659 | 0.000282167 | 0.003386 | 0.002831567 | NOX4/KCNE4/TDGF1/ABCG5/SLC7A9/SLC2A2/CDHR5/ABCG8 |
| GO:0034364 | high-density lipoprotein particle | CC | 3/107 | 26/19659 | 0.000372063 | 0.004185712 | 0.003500332 | APOA2/APOM/APOA1 |
| GO:0072562 | blood microparticle | CC | 5/107 | 147/19659 | 0.001259131 | 0.013331972 | 0.011148959 | SERPINF2/APOA2/APOA1/SERPINC1/A1BG |
| GO:0031091 | platelet alpha granule | CC | 4/107 | 91/19659 | 0.001541554 | 0.015204799 | 0.012715124 | MMRN1/SERPINF2/THBS2/A1BG |
| GO:0005604 | basement membrane | CC | 4/107 | 92/19659 | 0.001604951 | 0.015204799 | 0.012715124 | TNC/COL8A1/NID2/PTN |
| GO:0034774 | secretory granule lumen | CC | 7/107 | 321/19659 | 0.00187656 | 0.016889036 | 0.01412358 | SPP2/MMRN1/TTR/SERPINF2/SERPINA4/APOA1/A1BG |

*The clusterProfiler software was used for enrichment analysis of GO, and GO term with qvalue less than 0.05 was screened. ID: ID of GO entry;Description: Description information of GO entry;GeneRatio: Genes in the GO order annotated in differential genes, genes in the GO category annotated, and the ratio of the two;BgRatio: All genes annotated to this item, genes annotated to the GO item category, and the ratio of the two.Pvalue: P value of hypergeometric test;P.adjust: The P value corrected by multiple hypothesis testing;Qvalue: Q value;GeneID: the geneID annotated to the GO entry;Count: The number of genes added to the GO entry;Ont_type: Types of the GO entry, MF (molecular function), CC (cellular component), BP (biological process).
