# Supplementary material for: Prognostic analysis of tumor mutation burden and immune infiltration in hepatocellular carcinoma based on TCGA data
Source: Aging (Albany NY). 2021 Apr 4;13(8):11257–80. doi: 10.18632/aging.202811 (PMC8109113; doi:10.18632/aging.202811)
Supplement: Supplementary Table 4 [file aging-13-202811-s004.doc]

**Supplementary Table 4. Univariate Cox survival analysis of differentially expressed genes**

|  | **Var** | **HR** | **CI95.low** | **CI95.high** | **P.value** |
| --- | --- | --- | --- | --- | --- |
| 1 | FTCD | 0.861 | 0.809 | 0.916 | 3.00E-06 |
| 2 | SPP2 | 0.908 | 0.866 | 0.951 | 4.70E-05 |
| 3 | SLC2A2 | 0.886 | 0.833 | 0.943 | 0.000146 |
| 4 | DDTL | 0.769 | 0.67 | 0.883 | 0.000184 |
| 5 | A1BG | 0.89 | 0.835 | 0.95 | 0.000415 |
| 6 | CYP3A43 | 0.888 | 0.828 | 0.953 | 0.000997 |
| 7 | SLC22A25 | 0.901 | 0.843 | 0.963 | 0.002171 |
| 8 | IGSF23 | 0.9 | 0.841 | 0.963 | 0.002449 |
| 9 | SERPINF2 | 0.889 | 0.824 | 0.96 | 0.002576 |
| 10 | CYP3A5 | 0.882 | 0.813 | 0.958 | 0.002798 |
| 11 | AKR1D1 | 0.921 | 0.872 | 0.973 | 0.003208 |
| 12 | APOA1 | 0.92 | 0.868 | 0.975 | 0.004563 |
| 13 | SLC25A24 | 1.178 | 1.051 | 1.321 | 0.005017 |
| 14 | PFKP | 1.15 | 1.042 | 1.269 | 0.00554 |
| 15 | GNMT | 0.914 | 0.856 | 0.975 | 0.006304 |
| 16 | SLC38A3 | 0.894 | 0.825 | 0.969 | 0.006604 |
| 17 | LPA | 0.916 | 0.859 | 0.976 | 0.006936 |
| 18 | ACSL6 | 0.897 | 0.829 | 0.971 | 0.007364 |
| 19 | TTR | 0.916 | 0.859 | 0.978 | 0.008096 |
| 20 | LGALS3 | 1.154 | 1.036 | 1.285 | 0.0095 |
| 21 | SERPINC1 | 0.928 | 0.876 | 0.983 | 0.011334 |
| 22 | NPIPB15 | 0.917 | 0.855 | 0.984 | 0.015485 |
| 23 | DGAT2 | 0.89 | 0.809 | 0.978 | 0.015568 |
| 24 | SPDYC | 0.899 | 0.824 | 0.98 | 0.015629 |
| 25 | SEC16B | 0.864 | 0.767 | 0.973 | 0.016195 |
| 26 | LOX | 1.141 | 1.023 | 1.271 | 0.017461 |
| 27 | CES3 | 0.916 | 0.849 | 0.987 | 0.021872 |
| 28 | CYP2D6 | 0.924 | 0.863 | 0.99 | 0.024075 |
| 29 | DCN | 0.931 | 0.874 | 0.992 | 0.027783 |
| 30 | EXOC3L4 | 0.91 | 0.833 | 0.994 | 0.035891 |
| 31 | TMEM45A | 1.082 | 0.999 | 1.171 | 0.052394 |
| 32 | GPAM | 0.907 | 0.822 | 1.001 | 0.053225 |
| 33 | TUSC3 | 1.088 | 0.997 | 1.186 | 0.058424 |
| 34 | COL8A1 | 0.909 | 0.823 | 1.004 | 0.060633 |
| 35 | GRAMD1B | 1.079 | 0.996 | 1.167 | 0.061296 |
| 36 | DHRS2 | 0.954 | 0.907 | 1.003 | 0.064875 |
| 37 | ABCG5 | 0.928 | 0.858 | 1.005 | 0.065004 |
| 38 | TDGF1 | 0.947 | 0.894 | 1.004 | 0.067294 |
| 39 | NR1I3 | 0.937 | 0.87 | 1.009 | 0.085388 |
| 40 | EDA | 1.083 | 0.986 | 1.189 | 0.094739 |
| 41 | PTN | 0.926 | 0.842 | 1.018 | 0.111679 |
| 42 | CECR2 | 0.933 | 0.855 | 1.019 | 0.123032 |
| 43 | C17orf51 | 1.107 | 0.971 | 1.262 | 0.127011 |
| 44 | APOA2 | 0.954 | 0.894 | 1.018 | 0.156322 |
| 45 | POSTN | 1.051 | 0.979 | 1.127 | 0.169164 |
| 46 | SHBG | 0.948 | 0.877 | 1.025 | 0.177522 |
| 47 | APCDD1 | 1.043 | 0.979 | 1.11 | 0.190227 |
| 48 | MMRN1 | 1.079 | 0.961 | 1.211 | 0.197291 |
| 49 | CDHR5 | 0.956 | 0.892 | 1.026 | 0.214617 |
| 50 | SERPINA4 | 0.963 | 0.907 | 1.023 | 0.22333 |
| 51 | EPHA3 | 0.949 | 0.871 | 1.033 | 0.2276 |
| 52 | VCAN | 1.049 | 0.968 | 1.136 | 0.242745 |
| 53 | APOM | 0.949 | 0.868 | 1.037 | 0.246633 |
| 54 | DDC | 0.962 | 0.901 | 1.028 | 0.251606 |
| 55 | ARMCX3 | 1.06 | 0.958 | 1.173 | 0.255475 |
| 56 | CYP2D7 | 0.954 | 0.878 | 1.036 | 0.264842 |
| 57 | EDIL3 | 1.065 | 0.953 | 1.191 | 0.265837 |
| 58 | PMEPA1 | 1.057 | 0.956 | 1.168 | 0.280956 |
| 59 | TLX1 | 0.959 | 0.888 | 1.036 | 0.284198 |
| 60 | MLLT3 | 1.063 | 0.95 | 1.189 | 0.289037 |
| 61 | LUM | 0.971 | 0.919 | 1.026 | 0.294388 |
| 62 | ANTXR1 | 0.945 | 0.846 | 1.056 | 0.318933 |
| 63 | FABP1 | 0.973 | 0.922 | 1.027 | 0.321686 |
| 64 | ABCG8 | 0.968 | 0.908 | 1.032 | 0.322796 |
| 65 | THBS2 | 0.964 | 0.896 | 1.037 | 0.327795 |
| 66 | KCNE4 | 1.048 | 0.952 | 1.154 | 0.341388 |
| 67 | KIT | 1.056 | 0.938 | 1.188 | 0.369707 |
| 68 | GALNT7 | 1.055 | 0.935 | 1.19 | 0.385069 |
| 69 | HCN3 | 0.943 | 0.824 | 1.08 | 0.397709 |
| 70 | SULF1 | 1.036 | 0.953 | 1.127 | 0.404522 |
| 71 | ENPP7 | 0.969 | 0.9 | 1.044 | 0.407024 |
| 72 | CLIP2 | 1.05 | 0.931 | 1.183 | 0.429032 |
| 73 | COL1A1 | 1.032 | 0.945 | 1.126 | 0.486923 |
| 74 | PRKG1 | 0.955 | 0.835 | 1.091 | 0.496238 |
| 75 | ADAMTS12 | 1.044 | 0.921 | 1.184 | 0.499531 |
| 76 | LTBP1 | 1.038 | 0.921 | 1.171 | 0.540316 |
| 77 | CCL15 | 0.972 | 0.887 | 1.065 | 0.541634 |
| 78 | MOGAT3 | 1.026 | 0.943 | 1.115 | 0.554245 |
| 79 | COL1A2 | 1.03 | 0.932 | 1.139 | 0.56275 |
| 80 | ENDOD1 | 1.042 | 0.902 | 1.202 | 0.577871 |
| 81 | TANC2 | 1.035 | 0.916 | 1.169 | 0.580407 |
| 82 | SLIT2 | 0.975 | 0.891 | 1.068 | 0.588867 |
| 83 | NOX4 | 1.043 | 0.888 | 1.225 | 0.612252 |
| 84 | NAT8 | 0.98 | 0.902 | 1.065 | 0.638091 |
| 85 | PRODH2 | 0.985 | 0.922 | 1.052 | 0.650215 |
| 86 | PANX2 | 1.02 | 0.937 | 1.11 | 0.654038 |
| 87 | LIMCH1 | 1.029 | 0.906 | 1.168 | 0.661305 |
| 88 | COL12A1 | 0.978 | 0.871 | 1.097 | 0.70064 |
| 89 | RHBG | 0.992 | 0.948 | 1.039 | 0.738703 |
| 90 | SLC7A9 | 0.985 | 0.899 | 1.078 | 0.739155 |
| 91 | NID2 | 1.016 | 0.916 | 1.126 | 0.766298 |
| 92 | COL3A1 | 1.014 | 0.923 | 1.114 | 0.771318 |
| 93 | SEMA6D | 0.983 | 0.87 | 1.111 | 0.78414 |
| 94 | CDH11 | 1.013 | 0.921 | 1.114 | 0.795145 |
| 95 | SCD5 | 1.012 | 0.917 | 1.116 | 0.815046 |
| 96 | PCP4L1 | 0.992 | 0.923 | 1.066 | 0.82747 |
| 97 | CAPN12 | 0.99 | 0.9 | 1.09 | 0.838346 |
| 98 | PDE1A | 0.991 | 0.896 | 1.096 | 0.866003 |
| 99 | ARSE | 0.994 | 0.928 | 1.065 | 0.867212 |
| 100 | CRYBG3 | 1.007 | 0.924 | 1.097 | 0.879078 |
| 101 | PCDH18 | 1.009 | 0.893 | 1.14 | 0.882998 |
| 102 | OLFML1 | 0.992 | 0.886 | 1.111 | 0.894015 |
| 103 | HOXA13 | 1.003 | 0.953 | 1.056 | 0.897277 |
| 104 | TNC | 0.996 | 0.899 | 1.102 | 0.932645 |
| 105 | SSC5D | 0.997 | 0.905 | 1.097 | 0.946239 |
| 106 | DUSP4 | 0.996 | 0.887 | 1.119 | 0.949485 |
| 107 | AKT3 | 1.004 | 0.861 | 1.172 | 0.954934 |
| 108 | TC2N | 0.998 | 0.924 | 1.079 | 0.964651 |
| 109 | MOXD1 | 1 | 0.93 | 1.075 | 0.994951 |

*Unival single-factor Cox survival analysis was performed on 109 differentially expressed genes, and a P value of 0.05 was used as the screening criterion to obtain 30 genes with significant survival correlation.
